# The frequency of defective genomes in Omicron differs from that of the Alpha, Beta and Delta variants

**DOI:** 10.1038/s41598-022-24918-8

**Published:** 2022-12-29

**Authors:** Carolina Campos, Sergi Colomer-Castell, Damir Garcia-Cehic, Josep Gregori, Cristina Andrés, Maria Piñana, Alejandra González-Sánchez, Blanca Borràs, Oleguer Parés-Badell, Caroline Melanie Adombi, Marta Ibañez-Lligoña, Juliana Esperalba, Maria Gema Codina, Ariadna Rando-Segura, Narcis Saubí, Juan Ignacio Esteban, Francisco Rodriguez-Frías, Tomàs Pumarola, Andrés Antón, Josep Quer

**Affiliations:** 1 Liver Diseases-Viral Hepatitis, Liver Unit, Vall d’Hebron Institut de Recerca (VHIR), Vall d’Hebron Hospital Universitari, Vall d’Hebron Barcelona Hospital Campus, Passeig Vall d’Hebron 119-129, 08035 Barcelona, Spain; 2 Centro de Investigación Biomédica en Red de Enfermedades Hepáticas y Digestivas (CIBERehd), Instituto de Salud Carlos III, Av. Monforte de Lemos, 3-5, 28029 Madrid, Spain; 3 Biochemistry and Molecular Biology Department, Universitat Autònoma de Barcelona (UAB), Campus de La UAB, Plaça Cívica, 08193 Bellaterra, Spain; 4 Microbiology Department, Vall d’Hebron Institut de Recerca (VHIR), Vall d’Hebron Hospital Universitari, Vall d’Hebron Barcelona Hospital Campus, Passeig Vall d’Hebron 119-129, 08035 Barcelona, Spain; 5 Preventive Medicine, Vall d’Hebron Institut de Recerca (VHIR), Vall d’Hebron Hospital Universitari, Vall d’Hebron Barcelona Hospital Campus, Passeig Vall d’Hebron 119-129, 08035 Barcelona, Spain; 6 Biochemistry Department, Vall d’Hebron Institut de Recerca (VHIR), Vall d’Hebron Hospital Universitari, Vall d’Hebron Barcelona Hospital Campus, Passeig Vall d’Hebron 119-129, 08035 Barcelona, Spain; 7 Microbiology Department, Universitat Autònoma de Barcelona (UAB), Campus de La UAB, Plaça Cívica, 08193 Bellaterra, Spain; 8 Medicine Department, Universitat Autònoma de Barcelona (UAB), Campus de La UAB, Plaça Cívica, 08193 Bellaterra, Spain

**Keywords:** Evolution, Genetics, Microbiology, Molecular biology

## Abstract

The SARS-CoV-2 Omicron variant emerged showing higher transmissibility and possibly higher resistance to current COVID-19 vaccines than other variants dominating the global pandemic. In March 2020 we performed a study in clinical samples, where we found that a portion of genomes in the SARS-CoV-2 viral population accumulated deletions immediately before the S1/S2 cleavage site (furin-like cleavage site, PRRAR/S) of the *spike* gene, generating a frameshift and appearance of a premature stop codon. The main aim of this study was to determine the frequency of defective deletions in prevalent variants from the first to sixth pandemic waves in our setting and discuss whether the differences observed might support epidemiological proposals. The complete SARS-CoV-2 *spike* gene was deeply studied by next-generation sequencing using the MiSeq platform. More than 90 million reads were obtained from respiratory swab specimens of 78 COVID-19 patients with mild infection caused by the predominant variants circulating in the Barcelona city area during the six pandemic waves: B.1.5, B.1.1, B.1.177, Alpha, Beta, Delta, and Omicron. The frequency of defective genomes found in variants dominating the first and second waves was similar to that seen in Omicron, but differed from the frequencies seen in the Alpha, Beta and Delta variants. The changing pattern of mutations seen in the various SARS-CoV-2 variants driving the pandemic waves over time can affect viral transmission and immune escape. Here we discuss the putative biological effects of defective deletions naturally occurring before the S1/S2 cleavage site during adaption of the virus to human infection.

## Introduction

In a previous study performed in March 2020 including patients with mild and severe SARS-CoV-2 infection^1^, we reported that a minor population of viral genomes accumulated deletions at the S1/S2 cleavage site (PRRAR/S), generating a frameshift with appearance of a premature stop codon. This finding concurred with the results of a study published in 2020, where spike and S1 proteins were detected in serum of COVID19 patients^2^. We suggested a mechanism by which this event could reduce the severity of the infection and tissue damage without losing viral transmission capability.

With progression of the COVID-19 pandemic over time, new variants have arisen and some have been considered variants of concern (VOCs), such as the Alpha (B.1.1.7), Beta (B.1.351), Gamma (P.1), Delta (B.1.617.2)^3^, and more recently, Omicron (B.1.1.529) variant^4^. In our geographic area (Barcelona, Spain), B.1.5 and B.1.1 were the dominant variants during the first pandemic wave. These were replaced by B.1.177, which predominated in many European countries during the second wave. The Alpha variant replaced B.1.177 during the third wave, and later Delta outcompeted the other prevalent variants in regions where it appeared^5–7^. Omicron has quickly spread to become the world’s dominant variant. Within 4 weeks of its emergence, it was found in 100% of infected patients consulting in primary care centers of Barcelona city. A widespread explanation for this dominance is its higher transmissibility and likely higher resistance to the acquired immune response attained with the current COVID-19 vaccines and previous infections^8,9^. A surprising feature of this variant is that Omicron sequences are clustered away from the other SARS-CoV-2 genomes uploaded to GISAID^10–12^, which opens an interesting debate about the origin of Omicron.

Although data on the SARS-CoV-2 consensus sequence, point mutations, deletions, and insertions are continuously added to the GISAID repository, there is a lack of information on the composition of the viral quasispecies in clinical samples, and on changes in the quasispecies structure occurring over the consecutive pandemic waves. This information could be of value to understand the particular biology of SARS-CoV-2 and it might have implications regarding the epidemiology and origin of the virus and its variants.

Therefore, the aim of this study was to compare the type and frequency of defective deletions found in the SARS-CoV-2 spike gene in patients with mild infection caused by the most prevalent variants from the first to the sixth pandemic waves, including B.1.5, B.1.1, B.1.177, Alpha, Beta, Delta, and Omicron.

## Results

The analysis included 9 samples from patents with the B.1.5 variant, 8 with B.1.1, 11 with B.1.177, 9 with Alpha, 11 with Beta, 11 with Delta, and 19 with Omicron (Supplementary Table S1). In total, 91,526,555 reads (range 3814–620,216 reads per amplicon) were obtained from the 78 COVID-19-infected patients (Supplementary Table S2). Sequences from the B.1.5, B.1.1, B.1.177, Alpha, Beta, and Delta variants were uploaded to the GenBank Sequence Read Archive (SRA) database with BioProject accession number PRJNA788442, and sequences from Omicron with accession number SUB11151740.

Amplicon A78, running from nt 1905 to 2260 in the spike protein (aa636–aa753), is of particular interest because it includes the S1/S2 O-linked glycan residue polybasic TMPRSS2 cleavage site (aa685–aa686). We obtained coverages of 10,346 to 215,520 reads per amplicon (Supplementary Table S2) for A78, and it had the narrowest interquartile range (IQR) compared to the other spike regions (Supplementary Fig. S1). Comparison of coverage between amplicons showed no significant differences, with IQRs lower than 0.242 in all cases except for A82 and A84 (Supplementary Table S3).

Sixty-two of the 78 patients (79.5%) showed deleted haplotypes over 14 overlapping amplicons (Supplementary Figure S2A to S2G). In 28 of the 78 patients (35.9%), the deletions resulted in the appearance of a premature inframe stop codon, thus generating a defective genome (Supplementary Figure S3A to S3G). Specifically, we identified 49 defective deletions in these 28 patients, resulting in the loss of 2 (Δ2) to 100 (Δ100) nucleotides (Tables 1, 2, 3, 4, 5, 6 and 7). Deletions did not randomly accumulate along the *S* gene. Several were found in the same nucleotide position in different patients (Supplementary Figure S2A to S2G), even in some patients infected with different variants (Table 8). For example, deletion Δ817F-822L was found in three different patients (V69S13, V70S02, V70S03) with the B.1.5 variant (Table 1), but also in one patient (V69S03) with B.1.1 (Table 8).

**Table 1:** Variant B.1.5.

Deletion name	Amplicon	Δ (deleted nt positions)	aa	Δ size (nts deleted)	Patients	reads Δ	Total reads	Population frequency (%)
Δ244L–246R	A73	731–736	244L–246R	Δ6		482	62,756	0.77
Δ342F–343 N	A75	1024–1027	342F–343 N	Δ4		278	86,904	0.32
Δ427D–428D	A75	1281–1284	427D–428D	Δ4		279	86,904	0.32
Δ429F–444 K	A76	1285–1329	429F–444 K	Δ45		358	55,223	0.65
Δ456F–469S	A76	1368–1407	456F–469S	Δ40		187	102,058	0.18
Δ583E–585L	A77	1748–1755	583E–585L	Δ8		172	133,318	0.13
Δ656Y–670Y	A78	1980–2020	656Y–670Y	Δ13 to Δ41		2309	126,823	1.82
Δ817F–822L	A79	2451–2466	817F–822L	Δ11 to Δ16		1318	225,275	0.59
Δ852N–856 V	A80	2566–2578	852 N–856 V	Δ13		2788	67,62	4.12
Δ1107R–1130I	A82	3319–3389	1107R–1130I	Δ71		47	10,472	0.45
Δ1224L–1232I	A83	3670–3694	1224L–1232I	Δ25		157	52,409	0.30

**Table 2:** Variant B.1.1.

Deletion name	Amplicon	Δ (deleted nt positions)	aa	Δ size (nts deleted)	Patients	reads Δ	Total reads	Population frequency (%)
Δ108T–110L	A72	324–328	Δ108T–110L	Δ5		96	65,086	0.15
Δ244L–246R	A73–A74	731–738	Δ244L–246R	Δ5 to Δ6		509	218,599	0.23
Δ393T–394 N	A75	1177–1180	Δ393T–394 N	Δ4		441	87,224	0.51
Δ428D–433 V	A76	1284–1297	Δ428D–433 V	Δ14		449	78,022	0.58
Δ541F–546L	A77	1622–1638	Δ541F–546L	Δ17		540	82,513	0.65
Δ624I–629L	A77	1871–1887	Δ624I–629L	Δ17		458	82,513	0.56
Δ639F–646L	A78	1929–1948	Δ639F–646L	Δ20		139	66,033	0.21
Δ660I–670Y	A78	1991–2020	Δ660I–670Y	Δ28 to Δ29		275	119,349	0.23
Δ752L–753L	A79	2256–2259	Δ752L–753L	Δ4		148	67,171	0.22
Δ817F–822L	A79	2451–2466	Δ817F–822L	Δ16		313	67,171	0.47
Δ1262E–1263P	A84	3785–3788	Δ1262E–1263P	Δ4		40	21,1	0.19

**Table 3:** Variant B.1.177.

Deletion name	Amplicon	Δ (deleted nt positions)	aa	Δ size (nts deleted)	Patients	reads Δ	Total reads	Population frequency (%)
Δ21R–26P	A71	61–77	Δ21R–26P	Δ20	V75S13	133	97,697	0.14
Δ108T–118L	A72	324–352	Δ108T–118L	Δ5 to Δ29	V75S13–V77S12–V77S13	1036	306,998	0.34
Δ178D–179L	A73	533–536	Δ178D–179L	Δ4	V75S13	209	92,963	0.22
Δ244L–245H	A73–A74	731–734	Δ244L–245H	Δ4	V75S13	227	158,379	0.14
Δ276L–277L	A74	826–830	Δ276L–277L	Δ5	V75S13	102	65,416	0.16
Δ341V–342F	A75	1022–1025	Δ341V–342F	Δ5	V77S12–V77S13	742	253,541	0.29
Δ355V–361F	A75	1064–1083	Δ355V–361F	Δ20	V75S13	107	87,265	0.12
Δ393T–394 N	A75	1117–1180	Δ393T–394 N	Δ4	V77S12	196	124,546	0.16
Δ429F–438S	A76	1286–1313	Δ429F–438S	Δ28	V77S13	315	121,594	0.26
Δ656Y–670Y	A78	1980–2021	Δ656Y–670Y	Δ10 to Δ41	V71S10–V71S13–V75S13–V75S14–V77S12–V77S13	9813	407,468	2.02
Δ810S–822L	A79	2430–2466	Δ810S–822L	Δ11 to Δ33	V75S13–V77S13	848	184,691	0.46
Δ852N–856 V	A80	2567–2580	Δ852N–856 V	Δ4 to Δ14	V70S14–V75S13	728	129,549	0.56
Δ966L–970F	A81	2900–2909	Δ966L–970F	Δ10	V75S13	98	76,386	0.13
Δ1079P–1088H	A82	3236–3264	Δ1079P–1088H	Δ29	V75S13–V75S08	313	166,995	0.19
Δ1092E–1097S	A82	3276–3289	Δ1092E–1097S	Δ18	V75S13	157	53,336	0.29

**Table 4:** Variant B.1.1.7 (Alpha).

Deletion name	Amplicon	Δ (deleted nt positions)	aa	Δ size (nts deleted)	Patients	reads Δ	Total reads	Population frequency (%)
Δ15C-18L	A71	45–54	15C–18L	Δ10	V70S05	115	48,748	0.24
Δ59F-60S	A72	176–180	59F–60S	Δ5	V70S05	172	105,558	0.16
Δ108T-110L	A72	324–328	108 T–110L	Δ5	V70S11	118	67,512	0.17
Δ378K-394 N	A75	1132–1180	378 K–394 N	Δ49	V70S05	85	53,698	0.16
Δ852N-856 V	A80	2567–2580	852 N–856 V	Δ14	V70S11	79	52,884	0.15

**Table 5:** Variant B.1.351 (Beta).

Deletion name	Amplicon	Δ (deleted nt positions)	aa	Δ size (nts deleted)	Patients	reads Δ	Total reads	Population frequency (%)
Δ342F-343 N	A75	1024–1027	342F–343N	Δ4	V70S04	147	81,902	0.18
Δ817F-822L	A79	2451–2466	817F–822L	Δ16	V70S04	110	86,327	0.13
Δ852N-856 V	A80	2567–2580	852N–856V	Δ14	V70S04	66	38,495	0.17
Δ1079P-1088H	A82	3236–3264	1079P–1088H	Δ29	V75S03	148	94,956	0.16
Δ1092E-1097S	A82	3276–3289	1092E–1097S	Δ14	V75S03	148	94,956	0.16

**Table 6:** Variant B.1.617.2 (Delta).

Deletion name	Amplicon	Δ (deleted nt positions)	aa	Δ size (nts deleted)	Patients	reads Δ	Total reads	Population frequency (%)
Δ108T-110L	A72	324–328	108T–110L	Δ5	V77S02	179	113,692	0.16
Δ131C-145Y	A72	393–433	131C–145Y	Δ41	V77S02	154	113,692	0.14
Δ457R-471E	A76	1369–1411	457R–471E	Δ34 to Δ41	V77S02	324	118,481	0.27
Δ624I-629L	A77	1871–1887	624I–629L	Δ17	V77S02	198	85,582	0.23
Δ818I-822L	A79	2453–2466	818I–822L	Δ8 to Δ14	V77S02	401	75,176	0.53
Δ852N-856 V	A80	2567–2580	852N–856V	Δ14	V77S02	275	117,538	0.23

**Table 7:** Variant B.1.1.529 (Omicron).

Deletion name	Amplicon	Δ (deleted nt positions)	aa	Δ size (nts deleted)	Patients	reads Δ	Total reads	Population frequency (%)
Δ110L	A72	329–330	Δ110L	Δ2	V100S01	20	4859	0.41
Δ246R–249L	A73	736–745	Δ246R–249L	Δ10	V100S06	1299	18,474	7.03
Δ346R	A75	1036–1037	Δ346R	Δ2	V100S04	23	10,104	0.23
Δ396Y–397A	A75	1187–1190	Δ396Y–397A	Δ4	V100S08	36	8578	0.42
Δ397A–398D	A75	1191–1192	Δ397A–398D	Δ2	V98S12	20	14,510	0.14
Δ474Q–475A	A76	1421–1422	Δ474Q–475A	Δ2	V100S08	190	32,540	0.58
Δ594G	A77	1779–1780	Δ594G	Δ2	V100S08	157	119,784	0.13
Δ640S–674Y	A78	1920–2021	Δ640S–674Y	Δ2 to Δ100	V98S06–V100S0 V98S12–V100S01	932	152,298	0.69
Δ805I	A79	2413–2414	Δ805I	Δ2	V100S08	187	99,604	0.19
Δ851F–873Y	A80	2564–2632	Δ851F–873Y	Δ53 o Δ69	V98S12	22	1239	1.76
Δ867A–894F	A80	2612–2693	Δ867A–894F	Δ82	V98S06	13	7126	0.18
Δ890L–912L	A80	2681–2748	Δ890L–912L	Δ68	V98S06	25	7126	0.35
Δ1007Y/H–1008 V	A81	3018–3019	Δ1007Y/H – 1008 V	Δ2	V100S08	144	84,466	0.17
Δ1028K	A81	3080–3081	Δ1028K	Δ2	V100S01	739	111,880	0.66
Δ1062F	A82	3185–3186	Δ1062F	Δ2	V100S08	284	122,901	0.23
Δ1075F–1076 T	A82	3225–3226	Δ1075F–1076 T	Δ2	V100S08	295	122,901	0.24
Δ1087A–1099G	A82	3260–3296	Δ1087A–1099G	Δ60	V100S08	385	122,901	0.31

**Table 8:** Population frequency of defective genomes per deleted amino acid position (Del/Def) and variant.

Del/Def	B.1.5	B.1.1	B.1.177	Alpha B.1.1.7	Beta B.1.351	Delta B.1.617.2	Omicron B.1.1.529
Δ15C–18L				0.24			
Δ21R–26P			0.14				
Δ59F–60S				0.16			
Δ108T–110/118L		0.15	0.34	0.17		0.16	0.41
Δ131C–145Y						0.14	
Δ178D–179L			0.22				
Δ244L–245H/246R	0.77	0.23	0.14				
Δ246R–249L							7.03
Δ276L–277L			0.16				
Δ341V–342F			0.29				
Δ342F–343N	0.32				0.18		
Δ355V–361F			0.12				
Δ346R							0.23
Δ378K–394N				0.16			
Δ393T–394N		0.51	0.16				
Δ396Y–397A							0.42
Δ397A–398D							0.14
Δ427D–428D	0.32						
Δ428D–433V		0.58					
Δ429F–438S	0.65		0.26				
Δ456F–469S	0.18						
Δ457R–471E						0.27	
Δ474Q–475A							0.58
Δ541F–546L		0.65					
Δ583E–585L	0.13						
Δ594G							0.13
Δ624I–629L		0.56				0.23	
Δ639F–646L		0.21					
Δ640S–674Y	**1.82**	**0.23**	**2.02**				**0.69**
Δ752L–753L		0.22					
Δ755Q–756Y							
Δ805I							0.19
Δ810S–822L			0.46				
Δ817F–822L	0.59	0.47			0.13		
Δ818I–822L						0.53	
Δ851N–856V/873Y	4.12		0.56	0.15	0.17	0.23	1.76
Δ867A–894F							0.18
Δ890L–912L							0.35
Δ966L–970F			0.13				
Δ1007Y/H–1008V							0.17
Δ1028K							0.66
Δ1062F							0.23
Δ1075F–1076T							0.24
Δ1079P–1088H			0.29				
Δ1087A–1099G							0.31
Δ1092E–1097S			0.29				
Δ1107R–1130I	0.45						
Δ1224L–1232I	0.30						
Δ1262E–1263P		0.19					

Del = deletion; Def = deletions that cause a defective genome. The bold indicates the region where defective deletions were prevalent in different patients infected with variants at the beginning of the pandemic and in Omicron.

Table 1, 2, 3, 4, 5, 6 and 7 Deletions found along the spike gene from amplicon A73 to A83. Population frequency was calculated as the number of reads with deletions per amplicon divided by the total number of reads per amplicon. Table 1 for variant B.1.5; 1B for B.1.1; 1C for B.1.177; 1D for Alpha (B.1.1.7); 1E for Beta (B.1.351); 1F for Delta (B.1.617.2); and 1G for Omicron (B.1.1.529). Reads Δ = number of reads showing a deletion.

Sixteen of the 28 (57.1%) patients with defective mutations belonged to the first and second waves of the pandemic (B.1.5, B.1.1, and B.1.177) (Supplementary Figs. S3A−SG), whereas only 2 in 9 (7.1%) of Alpha, 2 in 11 (7.1%) of Beta, and 1 in 11 (3.6%) of Delta patients showed defective genomes. However, in contrast to the small percentage of defective mutations seen in Alpha, Beta and Delta patients, the Omicron variant showed defective genomes in 7 of the 28 (25%) patients.

Comparison of variants according to the genomic location of the defective positions showed some differences. The predominant lineages in the first and second wave (B.1.5, B.1.1, and B.1.177), as well as Omicron in the sixth wave, showed 11 to 18 deleted genomic regions leading to defective particles along the whole *spike* gene, from amplicon A71 to amplicon A84 (Tables 1, 2, 3 and 7). However, patients infected with the Alpha, Beta and Delta variants showed sporadic defective genomes only in amplicons A71, A72, A75, A76, A77, A79, A80, and A82 (Tables 4, 5 and 6).

As to the relative frequency of the variants found (number of defective reads per amplicon divided by total number of reads per amplicon) in all patients and in the whole *spike* gene, the largest percentage of variants with defective genomes were seen in the first and second pandemic waves (Tables 1, 2 and 3) and in Omicron (Table 7). The most prevalent defective deletion was Δ246R-249L in 7.03% of Omicron patients (Table 7), Δ852N–856V in 4.12% of B.1.5 patients (Table 1), and Δ656Y-670Y in 2.02% of B.1.177 and 1.82% of B.1.5. All other defective deletions were present at frequencies below 1% (Tables 1, 2 and 3).

**Figure 1:**
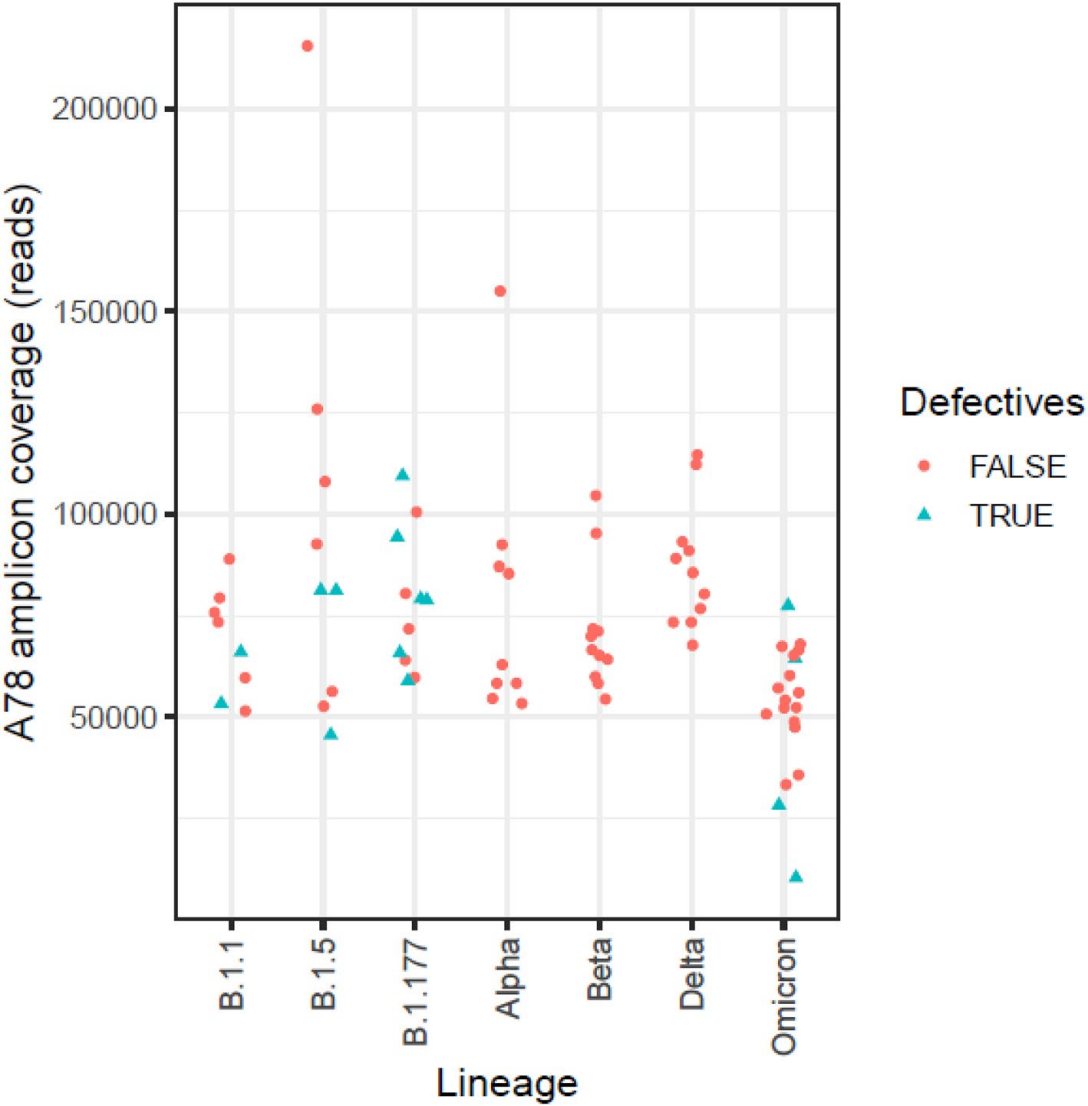
Amplicon A78 (nt 1905 to 2260, aa636-aa753) coverage per variant. Red dots represent reads with no defective haplotypes detected in amplicon A78 and blue triangles indicate reads with defective haplotypes in this position.

Of particular note, we found a significant presence of defective deletions in the Δ640S-674Y region (nt1920-2021) in amplicon A78 on the S1/S2 cleavage site in patients from the first and second waves and in Omicron patients, but not in Alpha, Beta and Delta patients (Table 2, Supplementary Fig. S4). The absence of defective genomes at this position in Alpha, Beta and Delta was not the result of inadequate sequencing coverage or number of patients studied (Supplementary Table S4). Defective genomes close to S1/S2 appeared in variants occurring at the beginning of the pandemic and in Omicron regardless of the number of sequences studied, whereas none of the Alpha, Beta or Delta patients showed any defective genomes in this sequence (Fig. 1). Moreover, although Omicron had the lowest sequencing coverage, it showed a pattern of defective deletions similar to those of B.1.1, B.1.5, and B.1.177.

Some specific defective deletions in the Δ640S–674Y fragment coincided in 2 or more patients infected by variants from the first and second waves. For example, the Δ656Y–670Y deletion was found in 3 patients with B.1.5 and 6 with B.1.177 (Tables 1, 2 and 3). Furthermore, Δ640S–674Y deletions were found in 4 patients with Omicron (Table 7). However, no deleted genomes in patients infected with the Alpha, Beta and Delta variants were detected in amplicon A78 (Tables 4, 5 and 6). Comparison of specific defective deletions between patients showed that deletions in the Δ640S–674Y region appeared only in patients with variants from the first and second pandemic waves (B.1.5, B.1.1, and B.1.177) at frequencies of 1.82%, 0.23%, and 2.02%, respectively, and in those with the Omicron variant (0.69%). This deletion was not detected in Alpha, Beta or Delta patients (Table 2) even though coverage was similar among all variants studied (Supplementary Fig. S4, Supplementary Table S4). However, we cannot exclude the presence of defective haplotypes with abundances below the 0.1% threshold.

The number, type, and frequency of deleted positions coincided in 22 of the 25 (88%) samples amplified in parallel using the ARTIC and N07^1^ protocols, ruling out bias caused by primer amplification (primers shown in Supplementary Table S5). In the remaining 4 samples, N07 detected deletions of 2 nucleotides that were not found with ARTIC, possibly because of the double PCR (RT-PCR-Nested) required for N07, which has a higher risk of introducing artefactual mutations, or the 2 to 5 times higher coverage in the N07 study. Interestingly, in 2 samples (patients V69S13 and V70S02), ARTIC was able to identify defective deletions that were not visualized with the N07 primers, even though ARTIC had lower coverage (81,248 and 45,575 reads) than N07 (242,697 and 240,491 reads) (Supplementary Table S6). In general, the ARTIC protocol identified a larger number of defective deletions, except for a 7-nt deletion in V70S12 and a 29-nt haplotype in V71S13, which were only seen using the N07 protocol. Our data show that results using ARTIC were concordant with those of N07, and that in the worst case, the N07 protocol might even underestimate the rate of defective deleted haplotypes.

## Discussion

**Figure 2:**
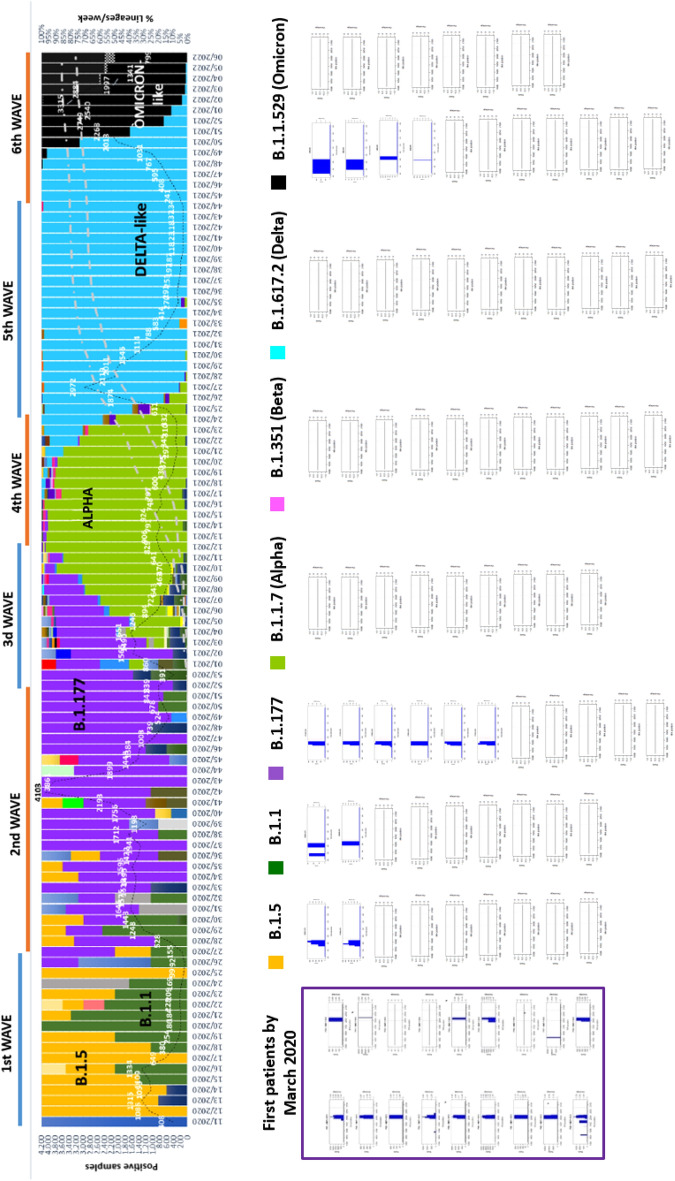
Lineage distribution per week and positive samples detected (discontinuous line) from March 2020 to February 2022 in Barcelona, Spain. Defective genomes are shown below the lineage distribution in bar plots starting from the first patients detected in March 2020: B.1.5 (yellow), B.1.1 (dark green), B.1.177 (violet), Alpha (light green), Beta (pink), Delta (blue), and Omicron (black). The bar plots below each variant indicate defective deletions in amplicon A78 in the 78 patients studied at the nucleotide level. The box located in the left corner are the defective bar plots related to patients in March 2020^1^. The x-axis provides the multiple alignment (MA) nucleotide positions and the amplitude of the deletions by subregions, and the y-axis shows the frequency of the deletion (percentage) on the right and the number of reads on the left. As no insertions causing defective genomes were observed, the MA positions correspond to S gene positions. Dashed lines indicate the S1/S2 (left) and S2’ (right) cleavage sites. Detailed bar plots for the 78 patients by amplicon are provided in supplementary figures (Supplementary Figures S2A−2G for deletions and 3A-3G for defective deletions).

The arrival of Omicron (B.1.1.529) to our geographic area has changed the profile of circulating SARS-CoV-2 variants, as it is now detected in 100% of infected patients in Barcelona city (Fig. 2). This predominance suggests that Omicron has biological advantages over the other variants: higher transmissibility and likely greater resistance to the immune response acquired by current COVID-19 vaccines or overcoming a previous SARS-CoV-2 infection^8,9^. The origin of Omicron is an open question. One hypothesis is that it could have evolved and emerged from a population undergoing little surveillance before being detected. Another proposal is that it may have resulted from viral evolution in patients with long-term persistent infection, a situation reported in clinical groups with a weak immune response, such as immunocompromised patients. A third hypothesis is that Omicron may have emerged by a cross-species jump from humans to nonhuman species, subsequently spilling back to humans^13,14^. Independently of its origin, whole-genome sequencing using next-generation techniques has shown that Omicron sequences are clustered far from the millions of SARS-CoV-2 genomes uploaded to GISAID. The present study points to an additional differentiating feature of Omicron: the pattern of mutations occurring at the S1/S2 cleavage site.

A higher presence of any variant in the human population as the pandemic progressed might reflect acquisition of a certain biological advantage over previous variants^15–17^. The scientific community has shown special interest in VOCs because of reported evidence indicating an impact on transmissibility and immunity attributed to multiple mutations in the receptor binding domain of the spike protein^6,7^. Another type of mutation involves deletions, which are a loss of nucleotides during the replication process that can cause a change in the correct reading frame. In a previous study^1^, we found that the early lineages showed deletions across the *spike* gene, some of them close to the S1/S2 *spike* cleavage site, which, in most cases, caused a frameshift and the appearance of a premature stop codon. Using a ribosome-profiling approach in samples from Germany, Finkel et al.^18^ described a deletion located in the furin-like cleavage site (TNSPRRAR, referred to in the present study as the S1/S2 cleavage site), affecting nucleotides 23,595 to 23,615, that was recurrently selected during passage of the original SARS-CoV-2 (EPI_ISL_406862) in Vero E6 cells^18^. In our analysis of 5,730,959 sequences from 78 patients, we did not find the exact deletion of that nucleotide region, which includes the amino acids PRRAR or HRRAR (Omicron). The deletions we identified occurred just before the cleavage site, specifically from nucleotide 23,555 (aa640S) to 23,580 (aa674Y). Nonetheless, the deletions found in both cases (naturally occurring and selected during Vero E6 viral cell culture) occurred in a small region of the spike, near or above the furin-like cleavage site, suggesting that this region is a hot spot for nucleotide deletions that could provide the virus with an evolutionary advantage. Postnikova et al.^19^ have suggested that the presence of two tandem CGG codons (the rarest in the SARS-CoV-2 genome), coding the two consecutive *R*s in the PRRAR region, could cause ribosomal pausing and even frameshifting. Translational pausing resulting from usage of an extremely rare dicodon, together with naturally occurring defective deletions close to S1/S2 that cause a frameshift with appearance of a new stop codon^1^, might suppress spike protein extension and lead to production of S1 free protein, a situation suggested in our previous study^1^. It is reasonable to postulate that the deletions and usage of rare codons indicate that this region is extremely important for the biology of the virus, enabling SARS-CoV-2 to readily infect humans. Free spike and truncated S1 protein, recently reported in plasma of severely ill COVID-19 patients, has been attributed to tissue damage resulting from severe disease^2^. In any case, this finding indicates that the complete spike protein and the truncated S1 form are soluble and can be secreted by infected cells, perhaps even in mild cases, as we suggested previously^1^.

Here we show that variants dominating the first (B.1.5, B.1.1) and second (B.1.177) pandemic waves had a large frequency of minority mutants with deletions causing defective genomes, whereas the Alpha, Beta and Delta variants, predominant in several regions of the world^5,20,21^, had a smaller presence of deleted genomes, suggesting that this situation may have favored their spread in the human population, overcoming other variants. Defective viral genomes occur in most, if not all, RNA viruses during infection^22^. Deep sequencing has shown that several species of defective genomes are generated, as was seen in samples from influenza virus-infected patients and cultures of metapneumovirus and measles virus^23–25^. Coronaviruses are not an exception^1,26–31^. It has been reported that defective genomes can interfere with wild-type viral infection, causing a reduction of virulence in vivo^32,33^, and there are many examples of defective genomes having an impact on human and animal health as a part of viral evolution and adaptation to the host^22^.

The Alpha, Beta and Delta variants appeared at a time when there was a high incidence of new infections, and they had to compete with other variants. The success of these lineages suggests that they had acquired a biological advantage to beat their competitors and dominate the pandemic wave. Our results show that Alpha, Beta, and Delta had no defective deletions in the *spike* region close to the S1/S2 cleavage site and much fewer defective deletions than the B.1.5, B.1.1, or B.1.177 lineages. A smaller presence of defective genomes might be associated with a greater presence of infectious viral particles, without affecting viral load (measured using the surrogate Ct value). With fewer defective genomes, there would be less circulating free SI and the putative competition of free S1 with infecting particles would decease or fail, with one outcome being greater infectivity. In addition, a significant reduction in defective genomes could be associated with longer duration of positive PCR testing and greater disease severity. It has been reported that the time interval from symptoms onset to viral load decrease is longer in infection by Delta than by other variants, and that Delta infection is associated with a higher risk of severe disease in unvaccinated people, and a greater need for remdesivir or corticosteroid treatment^34^. In their publication, the CDC concluded that Delta was more contagious than previous variants^35^ and a study from Ontario, Canada reported that there was a pronounced increase in hospitalization, ICU admission, and death during Delta variant dominance, particularly in unvaccinated people^36^.

Surprisingly, Omicron has shown a frequency of minority mutants with deletions more similar to variants dominating the first and second waves than to Alpha, Beta and Delta. Delta and its subvariants accounted for the vast majority of infections up to the end of 2021, and it was expected that one of these subvariants would ultimately predominate over others and become endemic and seasonal. In this scenario, the appearance of Omicron was unexpected and surprising, and its rise and dominance over Delta suggests that Omicron has higher fitness than other variants. The Omicron genome includes mutations that help the virus overcome the host’s immune response and spike gene mutations and deletions affecting the receptor binding domain and the S1/S2 cleavage site (HRRAR in Omicron) that increase host cell ACE2 receptor recognition. The explosion of Omicron, which at this time is the most prevalent variant all over the world, may also be explained by its tropism for the upper respiratory tract, which facilitates viral transmission^37^. Nonetheless, despite its dominance over Delta, Omicron subvariants BA.1 and BA.1.1 have not led to an increased severity of the infection (hospital admissions to ICU units) or mortality^38^.

In conclusion, the frequency of concomitant defective deletions close to the S1/S2 cleavage site was higher in dominant variants seen at the beginning of the pandemic than in the Alpha, Beta and Delta variants, suggesting that these mutations may contribute to adapting SARS-CoV-2 to human infectivity. The observation obtained here that the presence of mutations in Omicron is similar to that of variants at the beginning of the pandemic provides information that could be of value when studying the evolution of Omicron subvariants spreading among the human population. Our results concur with findings from previous studies indicating that the S1/S2 cleavage site is an important region for the biology of the virus, affecting the capability of SARS-CoV-2 to readily infect humans.

## Materials and methods

### Patients and methods

Naso/oropharyngeal swab samples were collected from laboratory-confirmed SARS-CoV-2 patients meeting the World Health Organization (WHO) definition^39^ admitted to the Vall d’Hebron University Hospital (HUVH) and from primary care centers in Barcelona. From April 2020 to December 2021, samples from patients carrying the most prevalent SARS-CoV-2 lineages over the 6 pandemic waves occurring in the Barcelona metropolitan area were randomly selected for deep-sequencing of the *spike* gene. B.1.5 and B.1.1 dominated the first wave (week 11/2020 to 26/2020), B.1.177 the second (week 27/2020 to 52/2020), B.1.1.7 (Alpha variant) the third (week 53/2020 to 13/2021), B.1.351 (Beta variant) the fourth (week 14/2021 to 25/2021), B.1.617.2 (Delta variant) the fifth wave (week 26/2021 to 43/2021), and B.1.1.519 (Omicron variant) the sixth wave (week 44/2021- ongoing).

The patients’ demographic data (sex and age) were obtained retrospectively. Clinical data, sample extraction data, cycle threshold (Ct) value (when measured), and symptoms are specified in Supplementary Table S1. Only samples with Ct values lower than 30 (with some exceptions) were included in the whole-genome sequencing weekly analysis^5^. Only samples from mild or asymptomatic patients were selected for study because this group showed the highest prevalence of deletions at the beginning of the pandemic^1^. Institutional review board of Clinical Research Ethics Committee (CEIm) Vall d’Hebron University Hospital approval was obtained(PR(AG)259/2020). The need for informed consent was waived by CEIm Vall d’Hebron University Hospital, as study used routinely collected for surveillance activities. All methods were performed in accordance with the relevant guidelines and regulations.

SARS-CoV-2 detection is described in the Supplementary Material for this study and in Andres et al.^1^. To sequence the whole *spike* gene, we mainly used ARTIC v3 primers including the 21,658 bp to 25,673 bp position, corresponding to the nCoV-2019_72 (A72) to nCoV-2019_84 (A84) overlapping amplicons (artic28-ncov2019/nCoV-2019.scheme.bed, ARTIC Network), and reformulated the ones that gave amplification problems due to deletions. *Spike* pair and impair primers were tested for efficacy and mixed in 2 different pools for the 2 posterior multiplexed PCRs. Each pool was optimized by adjusting primer concentration to obtain a balanced number of reads for each amplicon.

The bioinformatics analysis was done as reported by Andres et al.^1^, and the Wilcoxon test was used to compare Ct values between samples with and without emergent deletions. Pearson’s chi-square test was used to compare the emergence of deletions with sex or presence of COVID-19 disease symptoms (eg, high fever, anosmia, ageusia, persistent headache).

### Rationale for the bioinformatics analysis used

Conventional bioinformatics analyses can be carried out using several tools and platforms. In terms of sequence quality control, the analysis is performed with tools that can analyze various quality metrics and correct these if necessary. For example, in normal conditions, sequence trimming can be performed with the trimmomatic^40^ or fastp^41^ tools, which can remove low-quality sequences, nucleotides, primers, and adapters, among others. However, this approach could not be applied in our analysis, as reads would be shortened according to their quality and ultimately have different lengths, making proper analysis and comparison impossible.

With the use of conventional pipelines, once the reads are trimmed, you would map them against a reference to perform variant calling, where you would be able to see all changes. Several tools can be used for this type of analysis, such as bwa^42^, bowtie2^43^ or bbmap^44^ for alignment, and samtools or BCF tools^45^ for variant calling, among others. However, our aim was to detect deletions with specific characteristics according to the haplotypes present, which cannot be achieved using conventional pipelines, as this would result in the identification of all changes according to the reference.

In addition, analysis of the variants would lead to the consensus sequence, which was not the aim of our pipeline, as we were interested in studying the diversity of haplotypes.

### Ethics approval

The study was approved by the Institutional review board of Clinical Research Ethics Committee Vall d’Hebron University Hospital (CEIm), with reference PR(AG)259/2020. (https://vhir.vallhebron.com/en/institute/committee-and-commissions/clinical-research-ethics-committee-ceim).

## Supplementary Information

Supplementary Information.

## Data Availability

The datasets generated and/or analysed during the current study are available in the GenBank repository, GenBank Sequence Read Archive (SRA) database with BioProject accession number PRJNA788442, and sequences from Omicron with accession number SUB11151740.
