# Supplementary material for: The frequency of defective genomes in Omicron differs from that of the Alpha, Beta and Delta variants
Source: Sci Rep. 2022 Dec 29;12:22571. doi: 10.1038/s41598-022-24918-8 (PMC9799681; doi:10.1038/s41598-022-24918-8)
Supplement: Supplementary file 1 — Supplementary Information. [file 41598_2022_24918_MOESM1_ESM.docx]

**SUPPLEMENTARY MATERIAL**

**Methods**

**Detection of SARS-CoV-2**

SARS-CoV-2 detection was carried out by the following methods depending on their availability at the time of the analysis: a non-commercial, in-house PCR assay with primers and probes from the CDC 2019-nCoV PCR Panel and using the One-Step RT-PCR kit (Qiagen, Germany); commercial real-time RT-PCR assays, including the Allplex 2019-nCoV Assay (Seegene) and the Cobas SARS-CoV-2 Test (Roche Diagnostics); or TMA-based assays, including Procleix SARS-CoV-2 (Grifols) or Aptima SARS-CoV-2 (Hologic Inc.). Before performing the Qiagen and Seegene assays, total nucleic acids were extracted using the NucliSENS EasyMAG (bioMérieux, Marcy l’Etoile, France) or Microlab STARlet (Hamilton, CA, USA) system, depending on availability or the number of samples requiring extraction.

For comparisons of the cycle threshold (Ct) value, which is a good estimation of viral load, the TaqPath COVID-19 RT-PCR kit (Thermo Fisher Scientific, USA) was performed in all selected samples. This technique provides the Ct of three regions (*ORF1ab*, *spike*, and *N* genes). In this study we used the Ct provided from *N* gene quantitation, which is the value used in clinical reports. Clinical samples were maintained frozen (-80ºC) for further studies.

**SARS-CoV-2 whole spike sequencing**

Sample amplification was based on the ARTIC protocol (nCoV-2019 sequencing protocol V.3, Artic Network).[1] This method uses pair and impair primers to amplify overlapping amplicons (A) of the SARS-CoV-2 genome. We selected primers covering the complete *spike* region of SARS-CoV-2, from the 21658 bp to 25673 bp position, corresponding to the nCoV-2019_72 (A72) to nCoV-2019_84 (A84) overlapping amplicons (artic28-ncov2019/nCoV-2019.scheme.bed, Artic Network). *Spike* pair and impair primers were tested for efficacy and mixed in two different pools for the posterior two multiplexed PCRs. Each pool was optimized by adjusting primer concentration to that obtaining a balanced number of reads for each amplicon. Reverse transcription and PCR amplification were performed following the conditions established by the ARTIC protocol (nCoV-2019 sequencing protocol V.3, Artic Network).[1]

Purification of PCR products and library preparation were performed according to previously described methods.[2] Briefly, samples were normalized to 1.5 ng/µL, pooled in a single tube, and purified. Samples were then normalized to 4 nM before library preparation. Finally, the library was quantified by qPCR (KAPA Library Quantification Kit, Kapa Biosystems, Roche, Pleasanton, CA USA), denatured, and sequenced using the MiSeq platform (Illumina, San Diego, CA).

To investigate possible bias caused by the use of different primers, we amplified and deep-sequenced 26 samples from this study using both ARTIC primers and the N07 primers (Supplementary Table S3) used in our previous study.[2]

**Bioinformatic analysis: InDel study**

InDel analysis was done as reported by Andres et al.[2] Briefly, amplicons were reconstructed using FLASH, establishing a minimum of 20 overlapping bases and a maximum of 10% mismatches, while discarding low-quality reads. All reads with more than 5% of bases below a Phred Score of Q30 were filtered out. A fasta file was generated for each amplicon and each patient, and equal sequences were collapsed to obtain haplotypes with their corresponding frequencies. Low-abundance haplotypes (<0.1%) and those present only in the forward or reverse strand were discarded. Haplotypes common to both strands with frequencies above 0.1% (*consensus haplotypes*) were the basis of subsequent computations. Gaps were then removed, haplotypes were translated to amino acids, and stop haplotypes were trimmed after the stop. Single nucleotide deletions were excluded in this study, as they can be artifacts caused by PCR or sequencing errors. *Spike* gene deletions described to be a footprint of a particular variant, such as ΔH69-70 and ΔY144 for Alpha, ΔL242-244 for Beta, and Δ156-157 for Delta, were also excluded in the study.

Resulting amino acid haplotype sequences were realigned with MUSCLE (EMBL-EBI <https://www.ebi.ac.uk/Tools/msa/muscle/>), and whole genome consensus sequences were uploaded to the GISAID database (see codes in Table 1).[3] All computations were done in the R language and platform,[4] and in-house scripts were developed using the Biostrings,[5] Ape,[6] and ShortRead[7] packages.

**Statistical analysis**

The Wilcoxon test was used to determine whether there were differences in Ct values between samples with and without deletions. Pearson’s chi-square test was used to analyze associations between the presence or absence of deletions and patient sex or presence of COVID19 disease symptoms (eg, high fever, anosmia, ageusia, persistent headache).

**References**

1. nCoV-2019 sequencing protocol v3 (LoCost). https://www.protocols.io/view/ncov-2019-sequencing-protocol-v3-locost-bh42j8ye. Accessed 27 Oct 2021.

2. Andrés C, Garcia-Cehic D, Gregori J, Piñana M, Rodriguez-Frias F, Guerrero-Murillo M, et al. Naturally occurring SARS-CoV-2 gene deletions close to the spike S1/S2 cleavage site in the viral quasispecies of COVID19 patients. Emerg Microbes Infect. 2020;9:1900–11.

3. Elbe S, Buckland-Merrett G. Data, disease and diplomacy: GISAID’s innovative contribution to global health. Glob challenges (Hoboken, NJ). 2017;1:33–46.

4. Team RC. R: A language and environment for statistical computing. 2016.

5. Pages H, Aboyoun P, Gentleman R, DebRoy S. Biostrings:String objects representing biological sequences, and matching algorithms. R package 2.38.4. 2012.

6. Paradis E, Claude J, Strimmer K. APE: Analyses of Phylogenetics and Evolution in R language. Bioinformatics. 2004;20:289–90.

7. Morgan AM, Lawrence M, Anders S. Package “ShortRead.” Bioconductor Packag Maint. 2021.

**Supplementary Figures Legends**

**Supplementary Figure S1.** Box plot of the coverage per amplicon represented as logarithm of the reads.


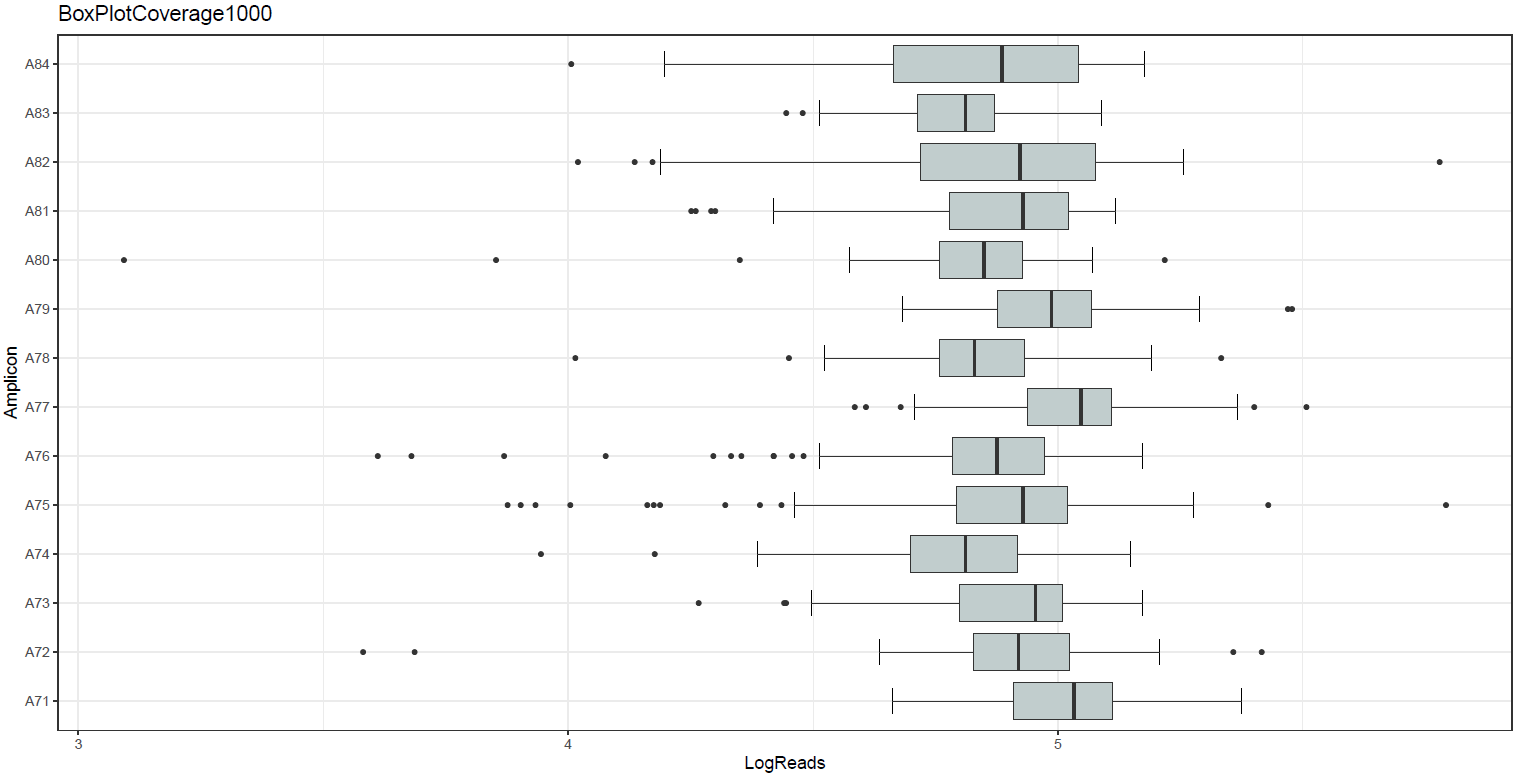


**Supplementary Figure S2A to 2G.** Bar plot of deletions per amplicon along the *spike* gene. Each bar plot found inside each table-box shows the deletion (blue columns), with the x axis providing the multiple alignment (MA) nucleotide positions and the amplitude of the deletion, and the y axis showing the frequency of the deletion (percentage) on the right and the number of reads on the left. As no insertions were observed, the MA positions correspond to *S* gene positions.


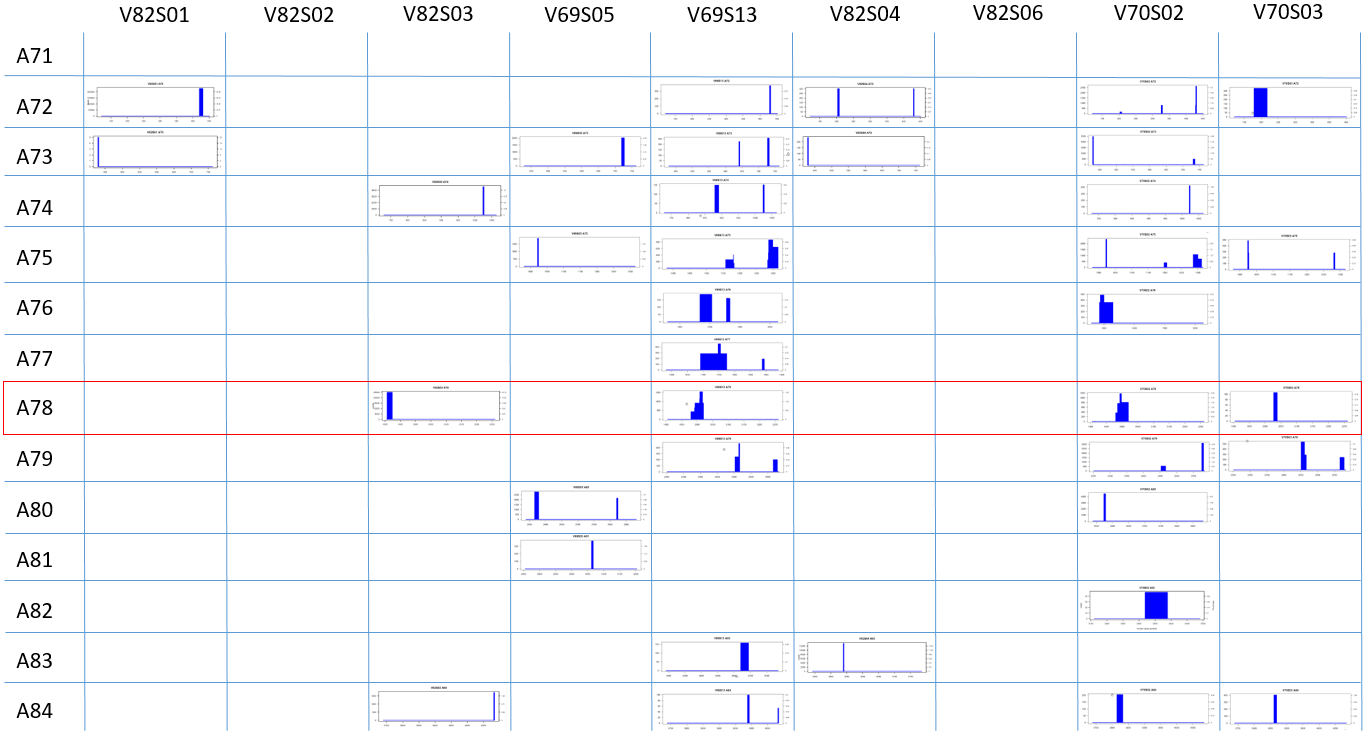
**Supplementary Figure S2A.** Variant B.1.5.


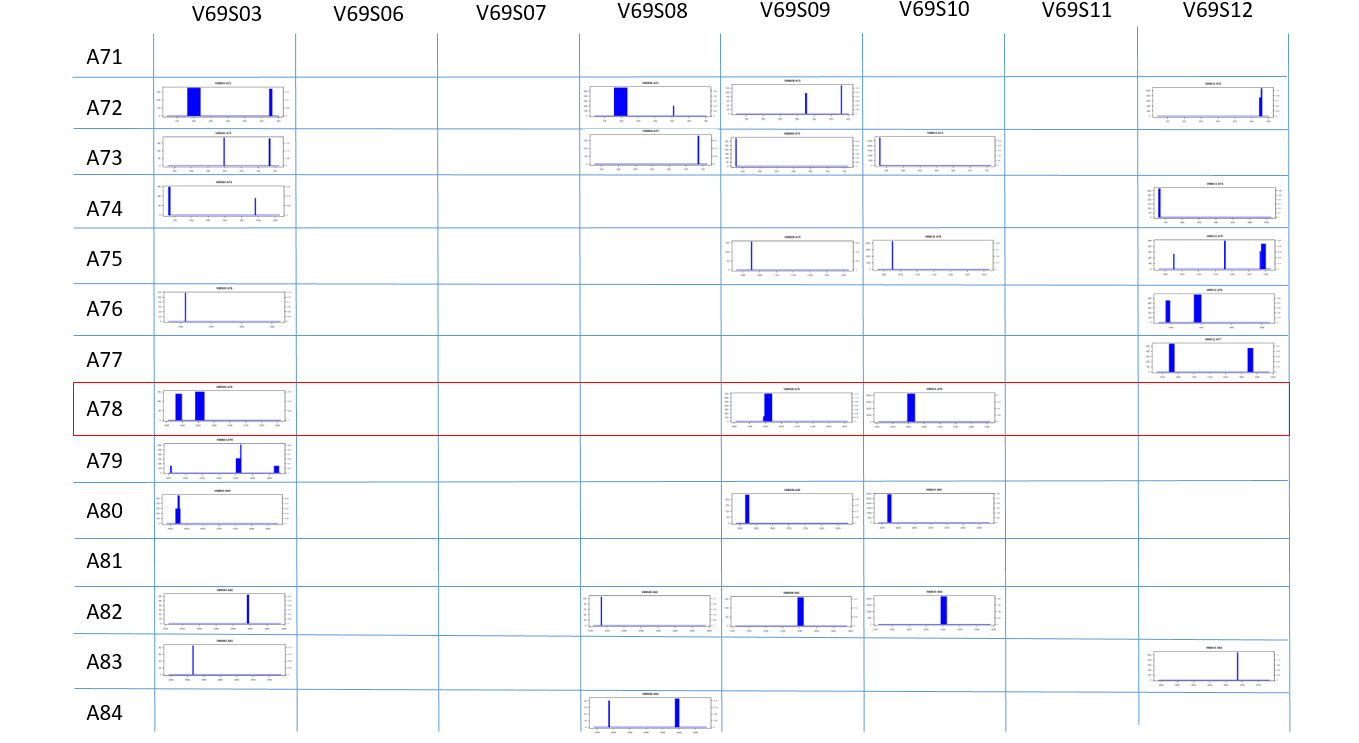
**Supplementary Figure S2B.** Variant B.1.1.

**
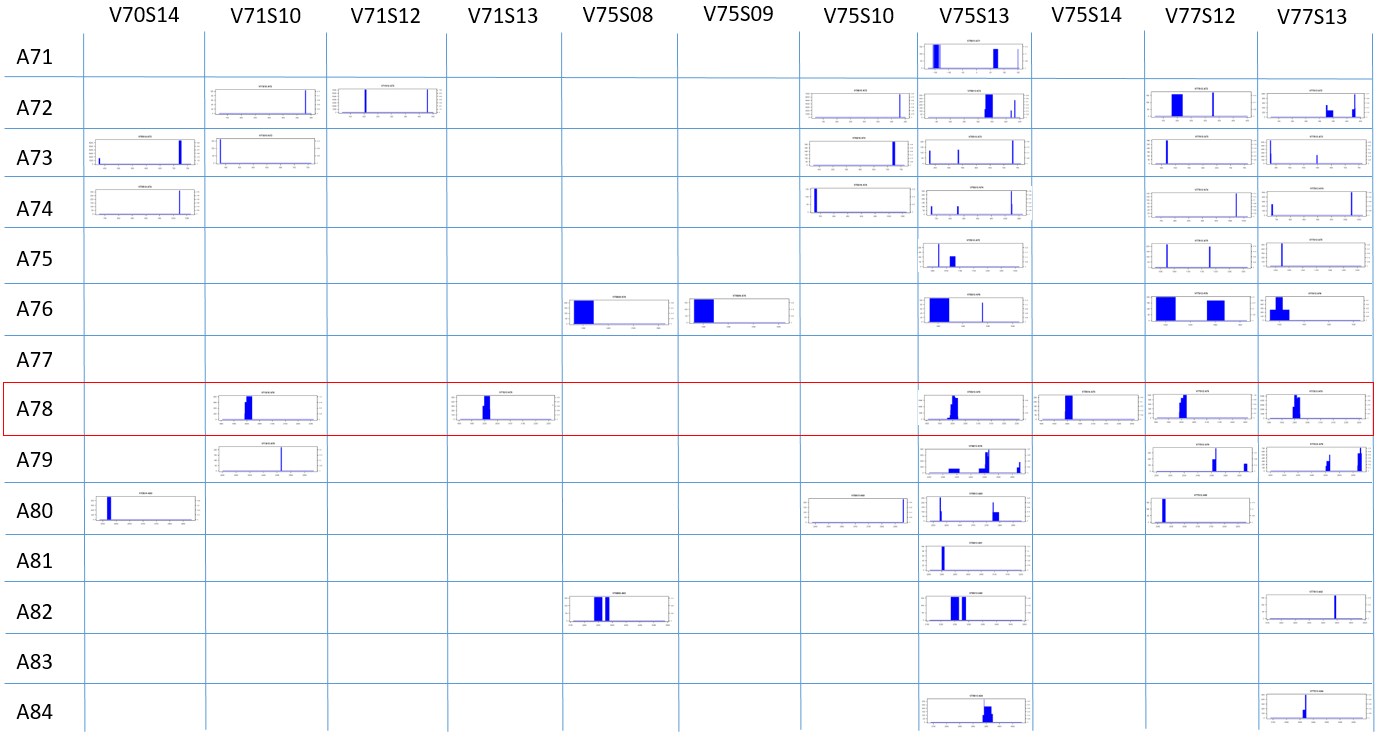
Supplementary Figure S2C.** Variant B.1.177.


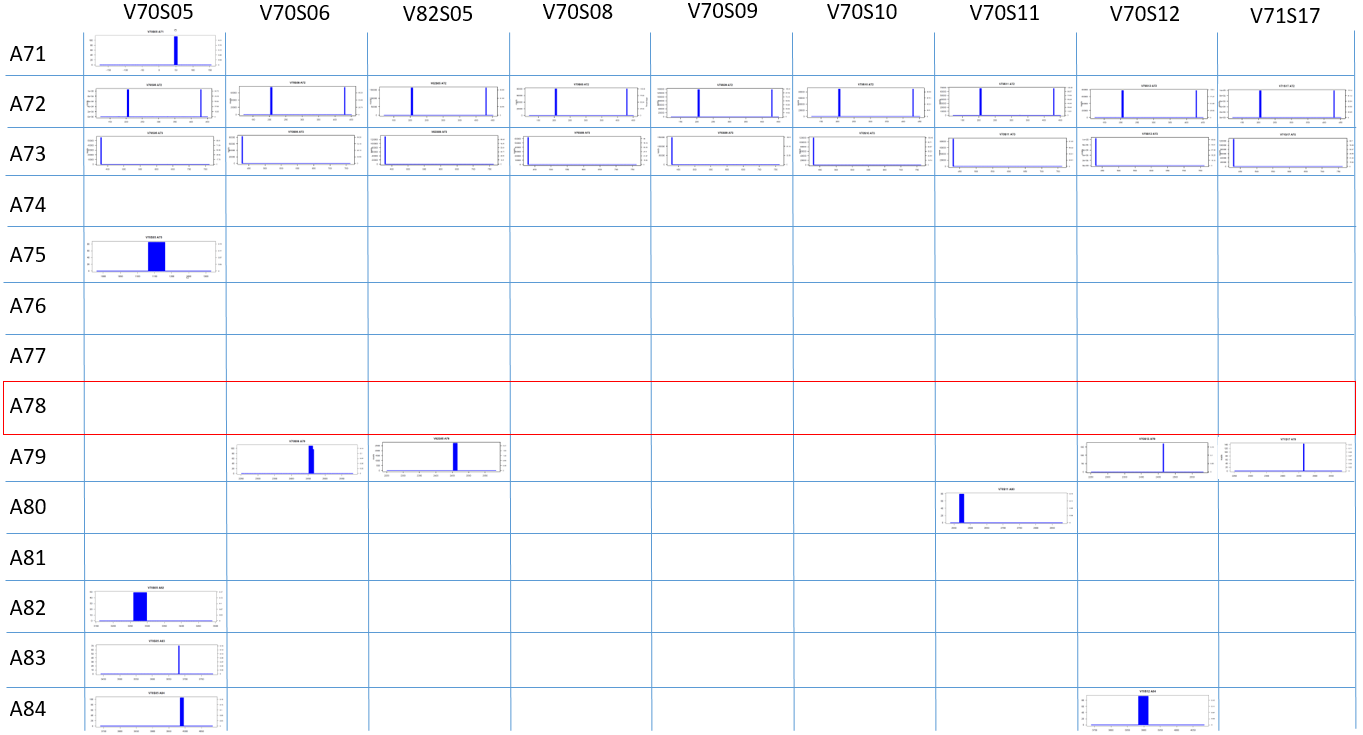
**Supplementary Figure S2D.** Variant B.1.17 (Alpha).


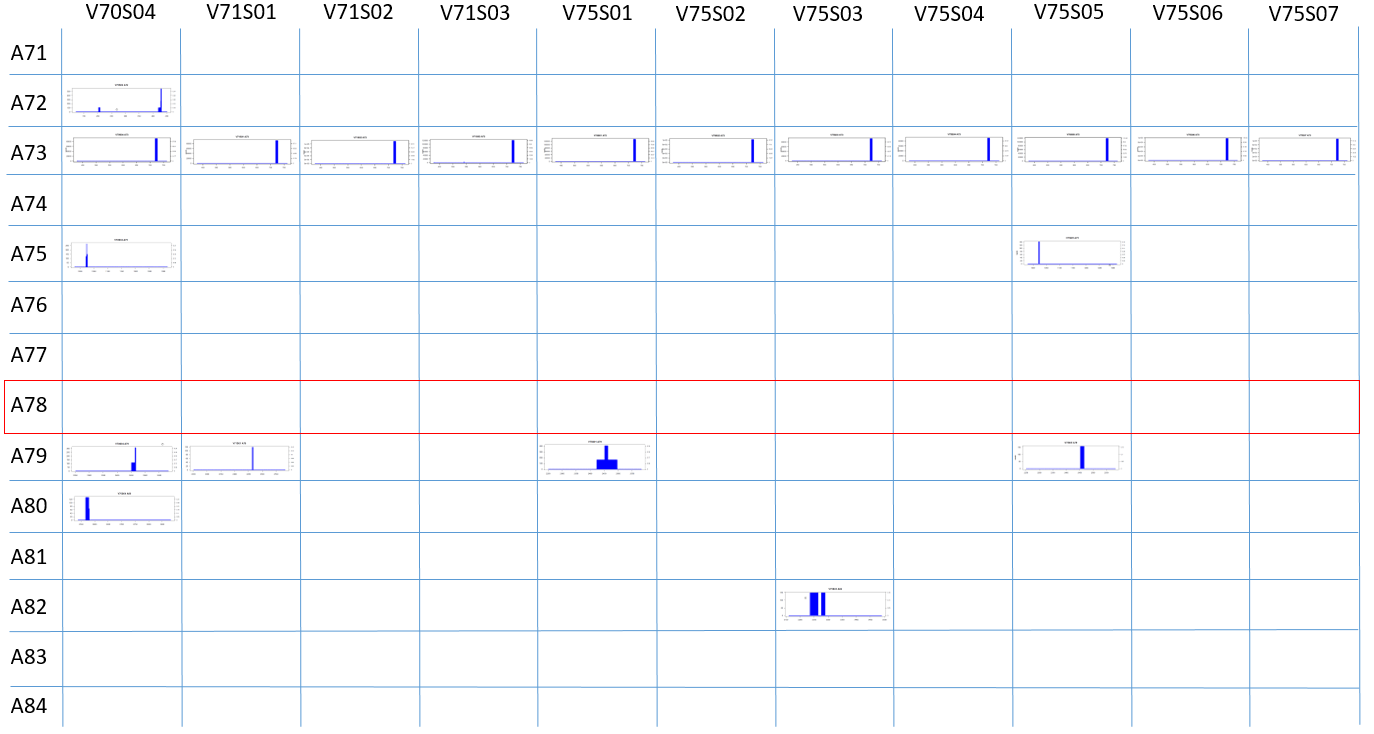
**Suppelementary Figure S2E.** Variant B.1.351 (Beta).


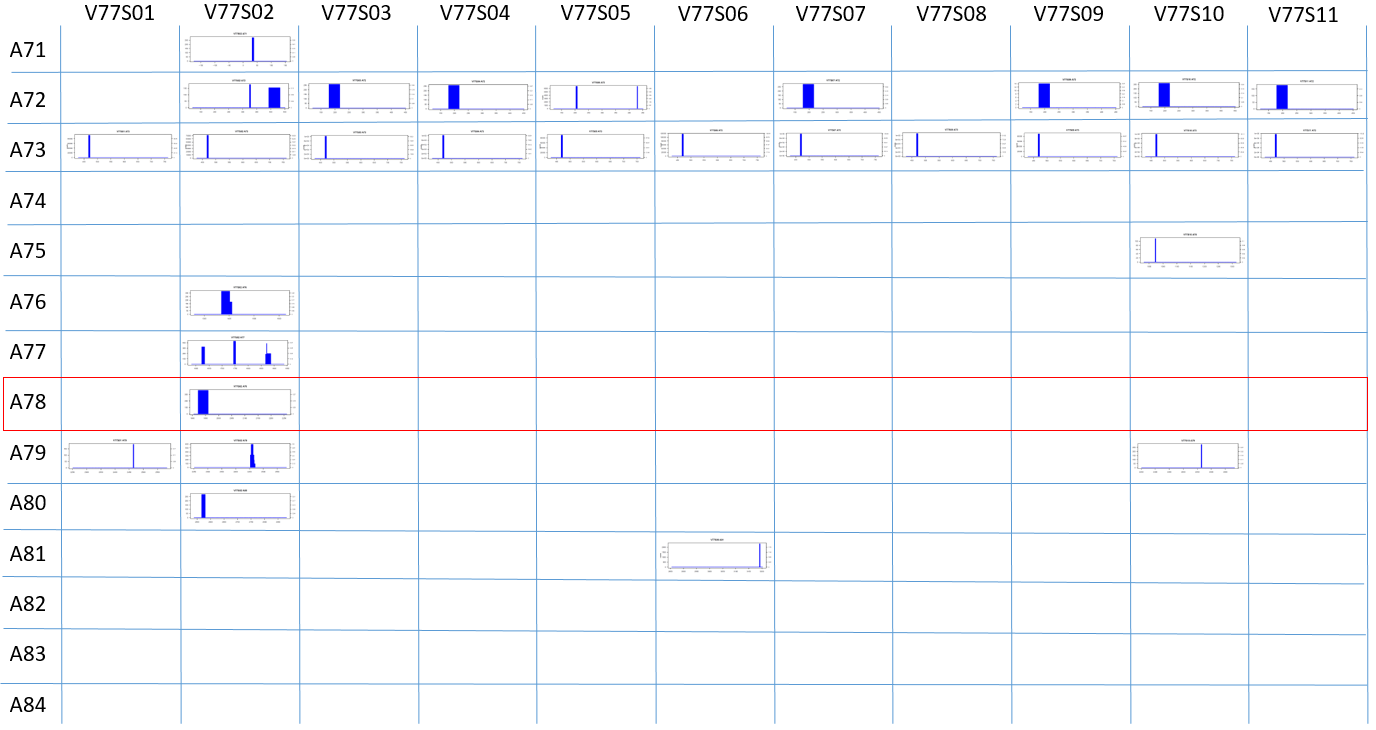
**Supplementary Figure S2F.** Variant B.1.617.2 (Delta).


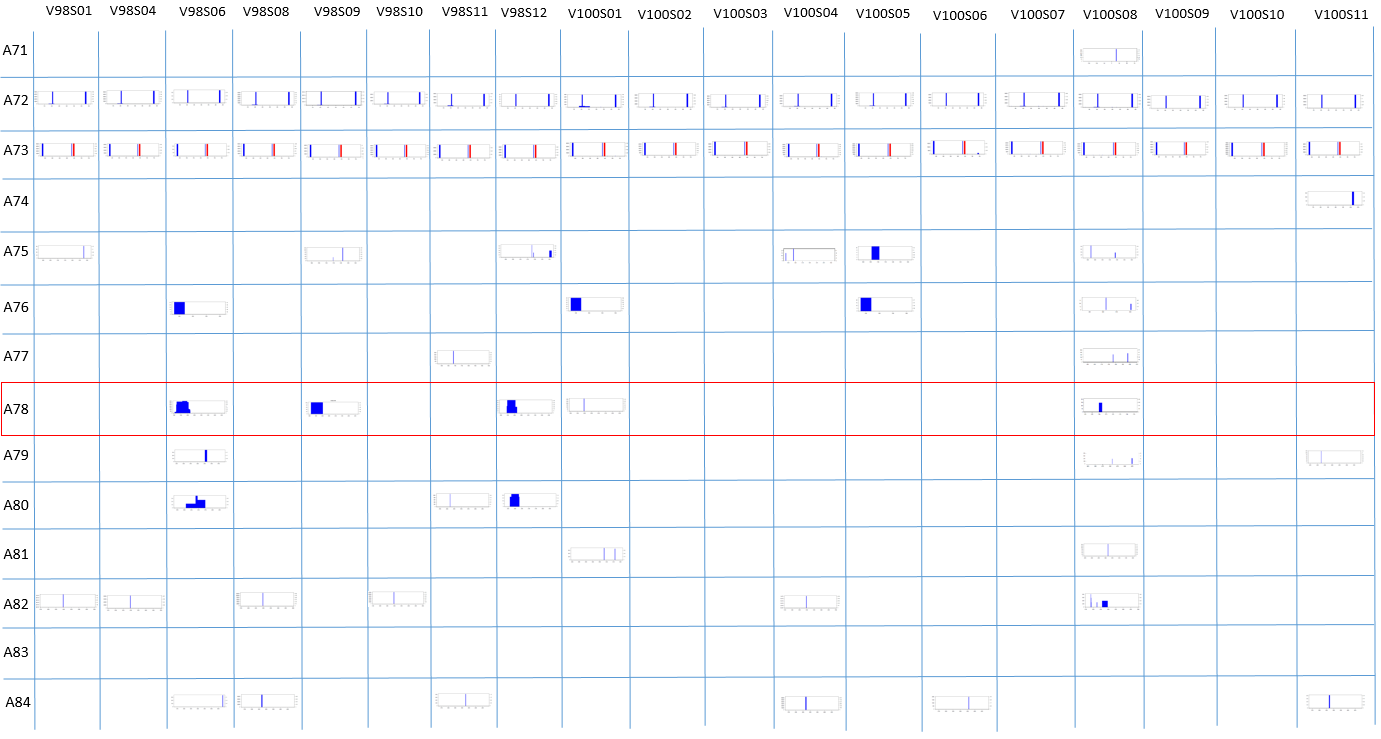
**Supplementary Figure S2G.** Variant B.1.1.529 (Omicron).

**Supplementary Figure S3A to 3G.** Bar plots with deletions that cause the appearance of a stop codon and that represent defective genomes.

**
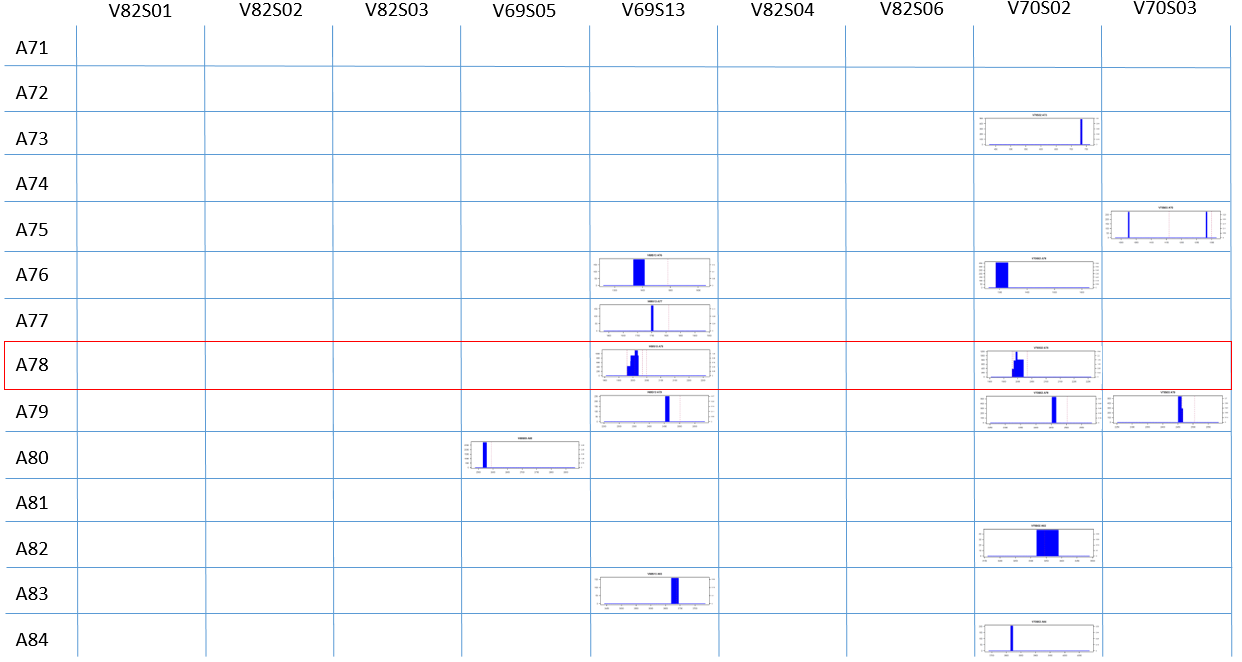
Supplementary Figure S3A**. Variant B.1.5.

**
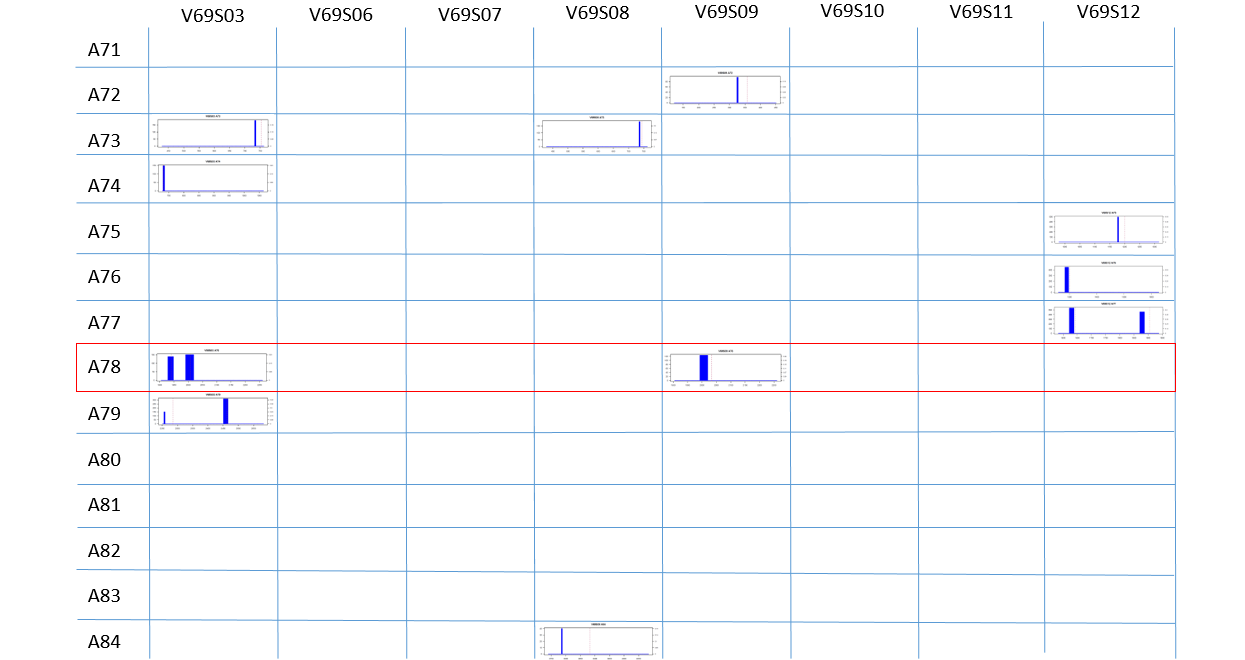
Supplementary Figure S3B.** Variant B.1.1.

**
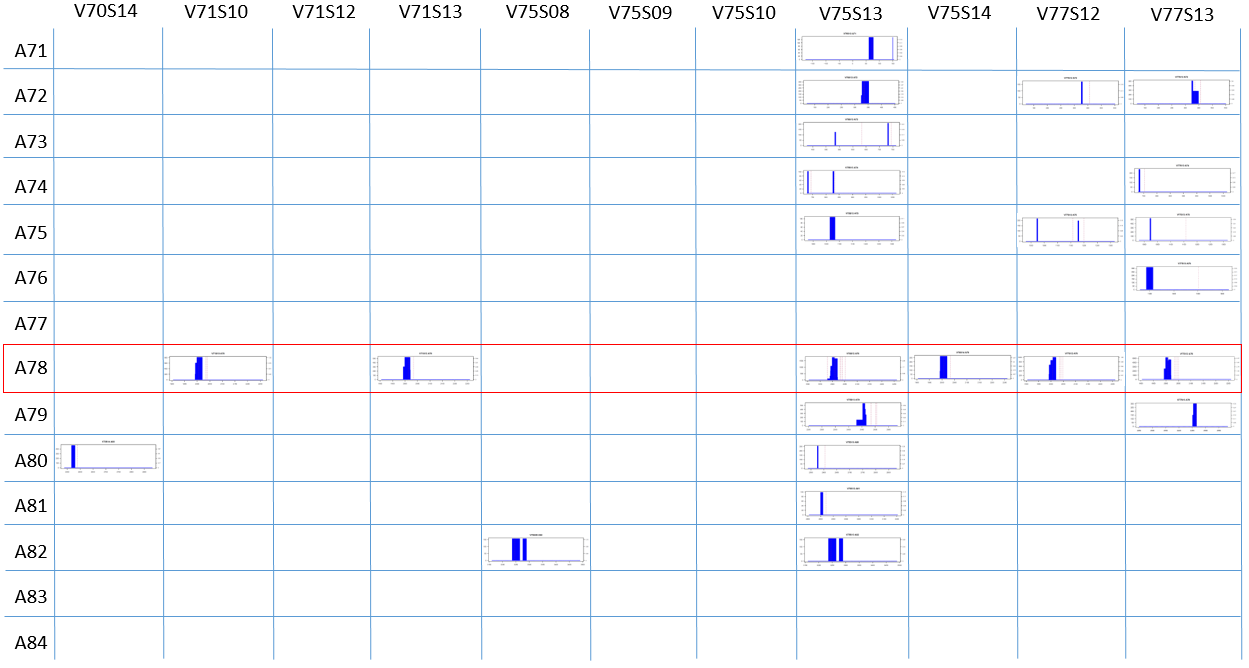
Supplementary Figure S3C**. Variant B.1.177.


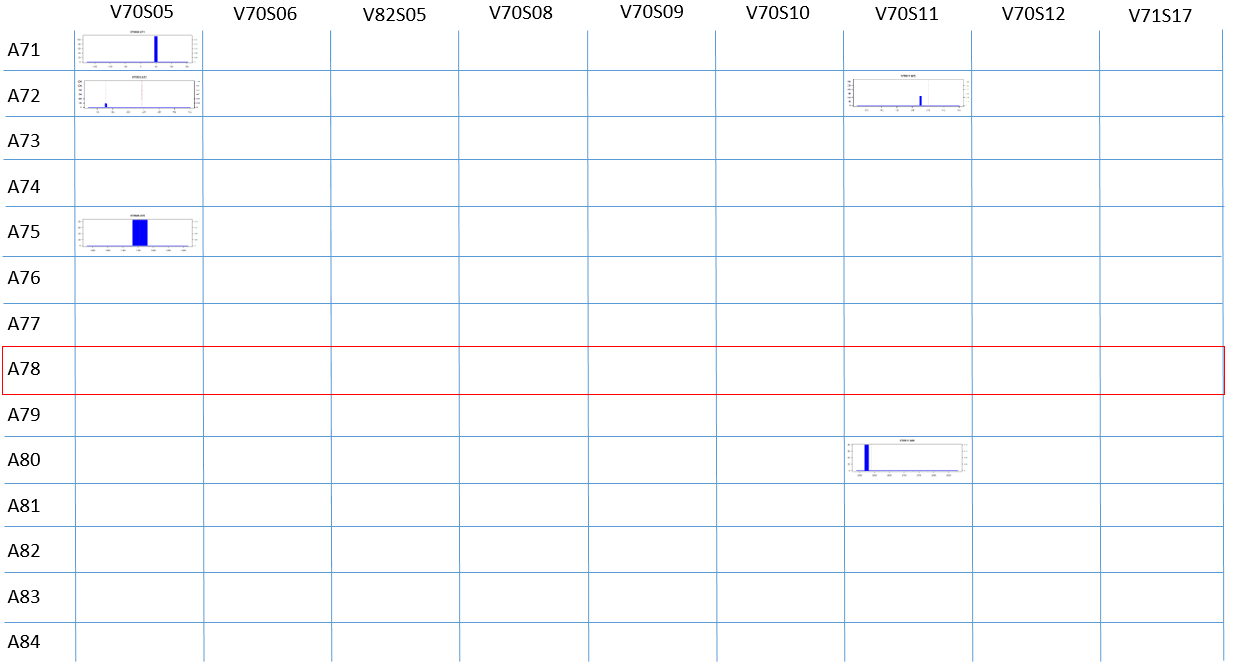
**Supplementary Figure S3D.** Variant B.1.1.7 (Alpha).


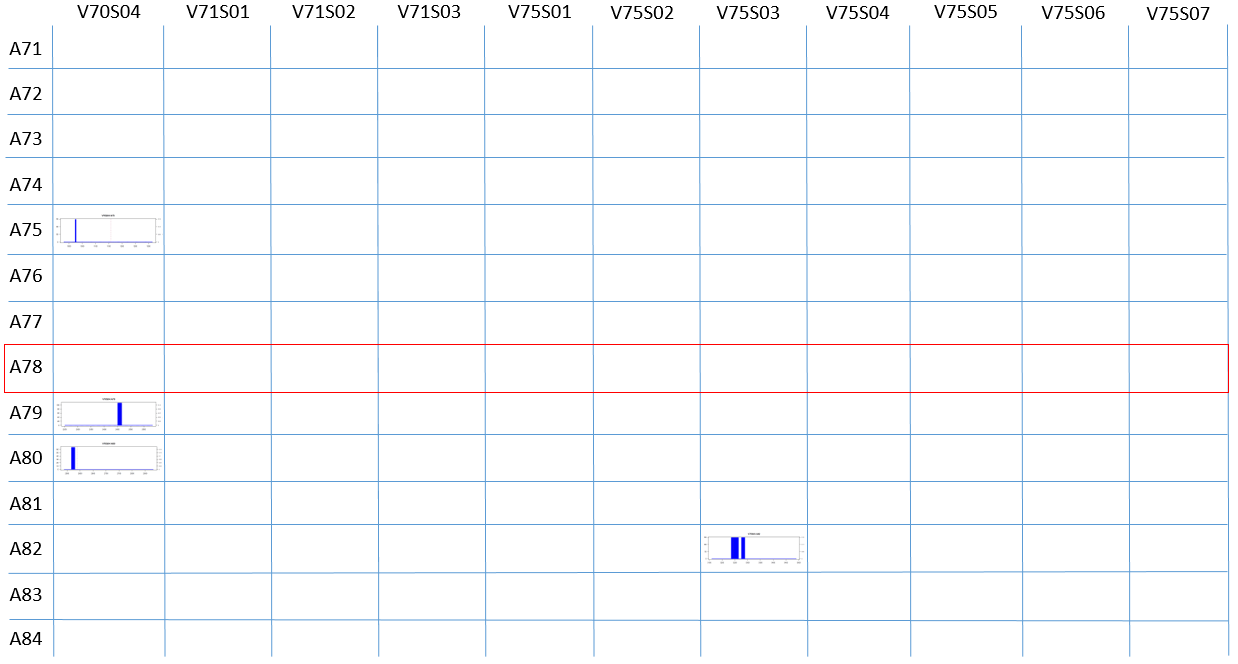
**Supplementary Figure S3E.** Variant B.1.351 (Beta).

**
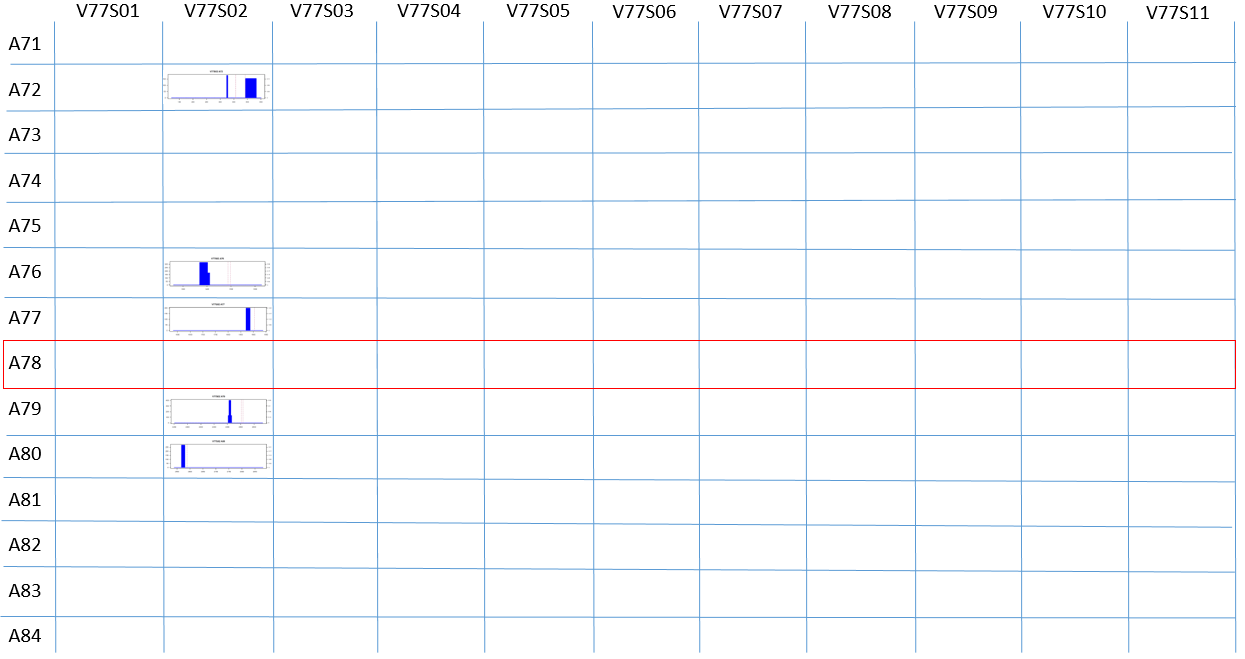
Supplementary Figure S3F.** Variant B.1.617.2 (Delta).

**
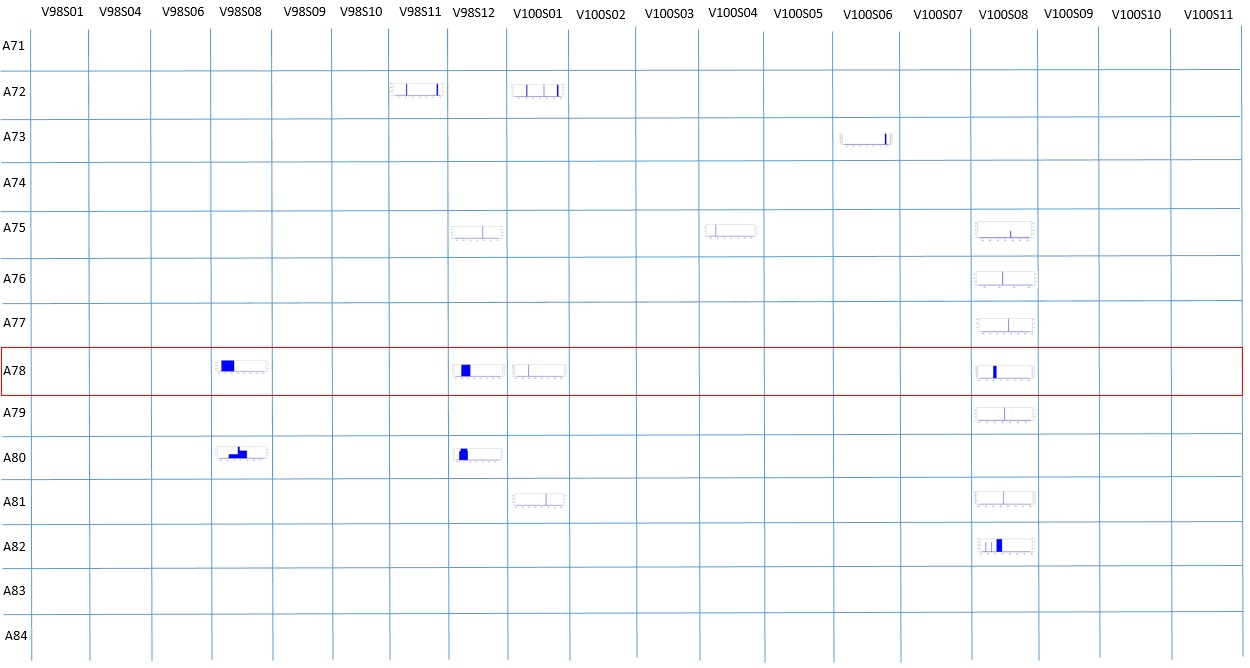
Supplementary Figure S3G.** Variant B.1.1.529 (Omicron).


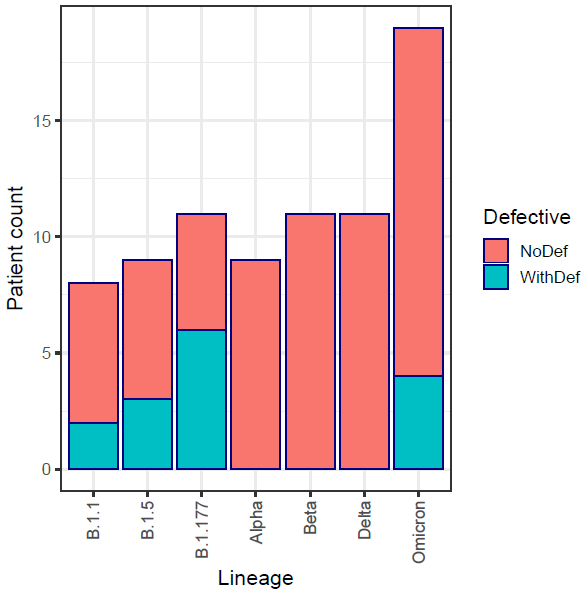
**Supplementary Figure S4.** Patients counting with no detected defective haplotypes (red color) and with defective haplotypes found (blue color) in amplicon A78 nt 1905 to 2260, aa636-aa753)

**Supplementary Table Legends**

**Supplementary Table S1.** Characteristics of patients. GISAID code refers to the uploaded complete consensus genome. Mild symptoms include fever, anosmia, ageusia, headache, etc. PC=primary care. H=Hospital. M=males, F=females, Ct=cycle threshold.

| **PATIENT ID** | **GISAID ID** | **LINEAGE** | **AGE** | **EXTRACTION DATE** | **Ct** | **TECHNIQUE** | **SYMPTOMS S/N** | **GENDER M o F** | **PRIMARY CARE Y/N** | **ENTRY HUVH Y/N** | **SAMPLE TYPE** |
| --- | --- | --- | --- | --- | --- | --- | --- | --- | --- | --- | --- |
| V82S01 | EPI_ISL_819299 | **B.1.5** | 60yo | 19/06/2020 | 33,996 | COBAS (TGT1) | N | F | N | N | naso/oropharyngeal exudate |
| V82S02 | EPI_ISL_819337 |  | 67yo | 04/05/2020 | 32,791 | COBAS (TGT1) | Y | M | N | N | naso/oropharyngeal exudate |
| V82S03 | EPI_ISL_819295 |  | 17yo | 29/04/2020 | 30,274 | COBAS (TGT1) | N | M | N | N | naso/oropharyngeal exudate |
| V69S05 | EPI_ISL_819335 |  | 33yo | 27/04/2020 | 28,412 | COBAS (TGT1) | Y | F | N | Y | naso/oropharyngeal exudate |
| V69S13 | EPI_ISL_819297 |  | 43yo | 30/04/2020 | 25,576 | COBAS (TGT1) | Y | F | Y | N | naso/oropharyngeal exudate |
| V82S04 | EPI_ISL_819344 |  | 48yo | 18/05/2020 | 29,209 | COBAS (TGT1) | Y | M | N | N | naso/oropharyngeal exudate |
| V82S06 | EPI_ISL_819350 |  | 78yo | 27/05/2020 | 26,914 | COBAS (TGT1) | Y | M | N | N | naso/oropharyngeal exudate |
| V70S02 | EPI_ISL_819298 |  | 77yo | 02/06/2020 | 28,159 | COBAS (TGT1) | N | M | N | N | naso/oropharyngeal exudate |
| V70S03 | EPI_ISL_819355 |  | 43yo | 04/06/2020 | 26,259 | COBAS (TGT1) | N | F | Y | N | naso/oropharyngeal exudate |
| V69S03 | EPI_ISL_819349 | **B.1.1** | 59yo | 26/05/2020 | 25,253 | COBAS (TGT1) | Y | F | N | N | naso/oropharyngeal exudate |
| V69S06 | EPI_ISL_819351 |  | 58yo | 28/05/2020 | 18,650 | COBAS (TGT1) | Y | M | Y | N | naso/oropharyngeal exudate |
| V69S07 | EPI_ISL_819352 |  | NA | 29/05/2020 | 29,335 | COBAS (TGT1) | N | F | Y | N | naso/oropharyngeal exudate |
| V69S08 | EPI_ISL_819353 |  | 14yo | 29/05/2020 | 19,199 | COBAS (TGT1) | Y | F | Y | N | naso/oropharyngeal exudate |
| V69S09 | EPI_ISL_819354 |  | 44yo | 03/06/2020 | 21,577 | COBAS (TGT1) | N | M | Y | N | naso/oropharyngeal exudate |
| V69S10 | EPI_ISL_819356 |  | 32yo | 05/06/2020 | 31,358 | COBAS (TGT1) | N | M | Y | N | naso/oropharyngeal exudate |
| V69S11 | EPI_ISL_819359 |  | 21yo | 11/06/2020 | 21,287 | COBAS (TGT1) | Y | F | Y | N | naso/oropharyngeal exudate |
| V69S12 | EPI_ISL_819360 |  | 32yo | 12/06/2020 | 26,849 | COBAS (TGT1) | Y | M | Y | N | naso/oropharyngeal exudate |
| V70S14 | EPI_ISL_1116590 | **B.1.177** | NA | 15/02/2021 | 29,349 | COBAS (TGT1) | N | M | Y | N | naso/oropharyngeal exudate |
| V71S10 | EPI_ISL_1116569 |  | 16yo | 17/02/2021 | 19,162 | COBAS (TGT1) | Y | M | Y | N | nasal swab |
| V71S12 | EPI_ISL_1116591 |  | 35yo | 15/02/2021 | 31,232 | COBAS (TGT1) | Y | F | Y | N | naso/oropharyngeal exudate |
| V71S13 | EPI_ISL_1116585 |  | 59yo | 16/02/2021 | 25,114 | COBAS (TGT1) | Y | F | Y | N | nasal swab |
| V75S08 | EPI_ISL_1669361 |  | 69yo | 08/04/2021 | 21,202 | COBAS (TGT1) | Y | F | Y | Y | naso/oropharyngeal exudate |
| V75S09 | EPI_ISL_1391101 |  | 13yo | 15/03/2021 | 26,236 | COBAS (TGT1) | N | M | Y | N | naso/oropharyngeal exudate |
| V75S10 | EPI_ISL_1391138 |  | 70yo | 17/03/2021 | 30,796 | COBAS (TGT1) | Y | M | Y | N | naso/oropharyngeal exudate |
| V75S13 | EPI_ISL_1797511 |  | 48yo | 16/04/2021 | 27,676 | COBAS (TGT1) | N | M | Y | N | naso/oropharyngeal exudate |
| V75S14 | EPI_ISL_1391158 |  | 45yo | 19/03/2021 | 16,700 | COBAS (TGT1) | Y | M | Y | N | naso/oropharyngeal exudate |
| V77S12 | EPI_ISL_1391140 |  | 91yo | 19/03/2021 | 25,078 | COBAS (TGT1) | Y | F | Y | N | naso/oropharyngeal exudate |
| V77S13 | EPI_ISL_1391141 |  | 93yo | 19/03/2021 | 26,439 | COBAS (TGT1) | N | F | Y | N | naso/oropharyngeal exudate |
| V70S05 | EPI_ISL_1116562 | **B.1.1.7** | 3yo | 17/02/2021 | 25,613 | COBAS (TGT1) | Y | M | Y | N | nasal swab |
| V70S06 | EPI_ISL_1116578 |  | 4yo | 18/02/2021 | 25,640 | COBAS (TGT1) | N | M | Y | N | nasal swab |
| V82S05 | EPI_ISL_1116564 |  | 3yo | 15/02/2021 | 28,386 | COBAS (TGT1) | Y | F | Y | N | nasal swab |
| V70S08 | EPI_ISL_1116558 |  | NA | 18/02/2021 | 11,470 | COBAS (TGT1) | N | M | Y | N | nasal swab |
| V70S09 | EPI_ISL_1116565 |  | 33yo | 15/02/2021 | 17,226 | COBAS (TGT1) | Y | F | Y | N | naso/oropharyngeal exudate |
| V70S10 | EPI_ISL_1116566 |  | 2yo | 15/02/2021 | 22,313 | COBAS (TGT1) | Y | F | Y | N | naso/oropharyngeal exudate |
| V70S11 | EPI_ISL_1116557 |  | 5yo | 16/02/2021 | 23,384 | COBAS (TGT1) | Y | F | Y | N | naso/oropharyngeal exudate |
| V70S12 | EPI_ISL_1116561 |  | 2yo | 18/02/2021 | 22,431 | COBAS (TGT1) | Y | F | Y | N | naso/oropharyngeal exudate |
| V71S17 | EPI_ISL_1116580 |  | 6yo | 16/02/2021 | 21,196 | COBAS (TGT1) | N | M | Y | N | naso/oropharyngeal exudate |
| V70S04 | EPI_ISL_1020573 | **B.1.351** | 38yo | 03/02/2021 | 22,448 | COBAS (TGT1) | Y | F | Y | N | naso/oropharyngeal exudate |
| V71S01 | EPI_ISL_918301 |  | 4yo | 19/01/2021 | 20,309 | COBAS (TGT1) | Y | M | Y | N | naso/oropharyngeal exudate |
| V71S02 | EPI_ISL_1080829 |  | 72yo | 10/02/2021 | 17,353 | COBAS (TGT1) | Y | M | Y | N | naso/oropharyngeal exudate |
| V71S03 | EPI_ISL_1080831 |  | 7yo | 12/02/2021 | 29,373 | COBAS (TGT1) | N | M | Y | N | nasal swab |
| V75S01 | EPI_ISL_2047723 |  | 4yo | 26/04/2021 | 25,058 | COBAS (TGT1) | Y | F | Y | N | naso/oropharyngeal exudate |
| V75S02 | EPI_ISL_2162254 |  | 4yo | 03/05/2021 | 19,736 | COBAS (TGT1) | N | M | Y | N | naso/oropharyngeal exudate |
| V75S03 | EPI_ISL_1797493 |  | 62yo | 14/04/2021 | 22,577 | COBAS (TGT1) | N | F | Y | N | naso/oropharyngeal exudate |
| V75S04 | EPI_ISL_1908823 |  | 1yo | 23/04/2021 | 14,673 | COBAS (TGT1) | Y | F | Y | N | naso/oropharyngeal exudate |
| V75S05 | EPI_ISL_2047794 |  | 67yo | 29/04/2021 | 24,098 | COBAS (TGT1) | Y | F | Y | N | naso/oropharyngeal exudate |
| V75S06 | EPI_ISL_2047795 |  | 67yo | 29/04/2021 | 14,445 | COBAS (TGT1) | Y | M | Y | N | naso/oropharyngeal exudate |
| V75S07 | EPI_ISL_1669413 |  | 7yo | 06/04/2021 | NA | COBAS (TGT1) | Y | M | Y | N | naso/oropharyngeal exudate |
| V77S01 | EPI_ISL_2284919 | **B.1.617.2** | 1yo | 12/05/2021 | 17,233 | COBAS (TGT1) | N | M | Y | N | naso/oropharyngeal exudate |
| V77S02 | EPI_ISL_2391926 |  | 26yo | 25/05/2021 | 26,663 | COBAS (TGT1) | Y | M | Y | N | naso/oropharyngeal exudate |
| V77S03 | EPI_ISL_2391913 |  | 43yo | 25/05/2021 | 16,119 | COBAS (TGT1) | Y | F | Y | N | naso/oropharyngeal exudate |
| V77S04 | EPI_ISL_2391912 |  | 44yo | 25/05/2021 | 13,349 | COBAS (TGT1) | N | M | Y | N | naso/oropharyngeal exudate |
| V77S05 | EPI_ISL_2458145 |  | 49yo | 25/05/2021 | 26,457 | COBAS (TGT1) | Y | F | Y | N | naso/oropharyngeal exudate |
| V77S06 | EPI_ISL_2458160 |  | 27yo | 26/05/2021 | 30,427 | COBAS (TGT1) | Y | F | Y | N | naso/oropharyngeal exudate |
| V77S07 | EPI_ISL_2458155 |  | 29yo | 26/05/2021 | 21,306 | COBAS (TGT1) | Y | F | Y | Y | naso/oropharyngeal exudate |
| V77S08 | EPI_ISL_2458152 |  | 46yo | 26/05/2021 | 19,245 | COBAS (TGT1) | Y | M | Y | N | naso/oropharyngeal exudate |
| V77S09 | EPI_ISL_2458146 |  | 47yo | 26/05/2021 | 14,529 | COBAS (TGT1) | Y | M | Y | N | naso/oropharyngeal exudate |
| V77S10 | EPI_ISL_2458140 |  | 43yo | 26/05/2021 | 18,714 | COBAS (TGT1) | N | M | Y | N | naso/oropharyngeal exudate |
| V77S11 | EPI_ISL_2493136 |  | 22yo | 01/06/2021 | 21,587 | COBAS (TGT1) | N | M | Y | N | naso/oropharyngeal exudate |
| V98S01 | EPI_ISL_8553063 | **B.1.1.529** | 24yo | 17/12/2021 | NA | COBAS (TGT1) | NA | F | Y | N | naso/oropharyngeal exudate |
| V98S04 | EPI_ISL_8553046 |  | 50yo | 17/12/2021 | NA | COBAS (TGT1) | NA | F | Y | N | naso/oropharyngeal exudate |
| V98S06 | EPI_ISL_8552995 |  | 23yo | 17/12/2021 | NA | COBAS (TGT1) | NA | F | Y | N | naso/oropharyngeal exudate |
| V98S08 | EPI_ISL_8553066 |  | 22yo | 17/12/2021 | NA | COBAS (TGT1) | NA | F | Y | N | naso/oropharyngeal exudate |
| V98S09 | EPI_ISL_8553061 |  | 52yo | 17/12/2021 | NA | COBAS (TGT1) | NA | F | Y | N | naso/oropharyngeal exudate |
| V98S10 | EPI_ISL_8553227 |  | 50yo | 16/12/2021 | NA | COBAS (TGT1) | NA | F | Y | N | naso/oropharyngeal exudate |
| V98S11 | EPI_ISL_8552957 |  | 34yo | 16/12/2021 | NA | COBAS (TGT1) | NA | F | Y | N | naso/oropharyngeal exudate |
| V98S12 | EPI_ISL_3047229 |  | 38yo | 15/12/2021 | NA | COBAS (TGT1) | NA | M | Y | N | naso/oropharyngeal exudate |
| V100S01 | EPI_ISL_8552968 |  | 24yo | 15/12/2021 | NA | COBAS (TGT1) | NA | F | Y | N | naso/oropharyngeal exudate |
| V100S02 | EPI_ISL_7597244 |  | 31yo | 15/12/2021 | NA | COBAS (TGT1) | NA | F | Y | N | naso/oropharyngeal exudate |
| V100S03 | EPI_ISL_855297 |  | 32yo | 15/12/2021 | NA | COBAS (TGT1) | NA | M | Y | N | naso/oropharyngeal exudate |
| V100S04 | EPI_ISL_8553020 |  | 36yo | 15/12/2021 | NA | COBAS (TGT1) | NA | M | Y | N | naso/oropharyngeal exudate |
| V100S05 | EPI_ISL_8553025 |  | 35yo | 15/12/2021 | NA | COBAS (TGT1) | NA | M | Y | N | naso/oropharyngeal exudate |
| V100S06 | EPI_ISL_8553259 |  | 28yo | 14/12/2021 | NA | COBAS (TGT1) | NA | M | Y | N | naso/oropharyngeal exudate |
| V100S07 | EPI_ISL_8553647 |  | 31yo | 14/12/2021 | NA | COBAS (TGT1) | NA | M | Y | N | naso/oropharyngeal exudate |
| V100S08 | EPI_ISL_8553024 |  | 57yo | 14/12/2021 | NA | COBAS (TGT1) | NA | F | Y | N | naso/oropharyngeal exudate |
| V100S09 | NA |  | NA | NA | NA | COBAS (TGT1) | NA | NA | Y | N | naso/oropharyngeal exudate |
| V100S10 | NA |  | NA | NA | NA | COBAS (TGT1) | NA | NA | Y | N | naso/oropharyngeal exudate |
| V100S11 | NA |  | NA | NA | NA | COBAS (TGT1) | NA | NA | Y | N | naso/oropharyngeal exudate |

**Supplementary Table S2.** Total number of reads per amplicon obtained from the 78 COVID-19-infected patients. NA indicates that amplification was unsuccessful and no amplicons were obtained during sequencing. Box highlighted in light orange <20000, orange >20000, <35000, green <10000 or NA, boxed cells >220000 reads.

| **GISAID ID** | **Llinatge** | **Columna1** | **A71** | **A72** | **A73** | **A74** | **A75** | **A76** | **A77** | **A78** | **A79** | **A80** | **A81** | **A82** | **A83** | **A84** | **total reads** |
| --- | --- | --- | --- | --- | --- | --- | --- | --- | --- | --- | --- | --- | --- | --- | --- | --- | --- |
| EPI_ISL_819299 | **B.1.5** | **V82S01** | 180171 | 228175 | NA | 67185 | 189362 | 65457 | 104521 | 215520 | 115423 | 165291 | NA | 122280 | NA | 23991 | 1477376 |
| EPI_ISL_819337 | **B.1.5** | **V82S02** | 236722 | 126471 | 129889 | 77333 | 620216 | NA | 251736 | 92577 | 294902 | 81140 | NA | 79512 | NA | 30151 | 2020649 |
| EPI_ISL_819295 | **B.1.5** | **V82S03** | 48909 | 100342 | 32383 | 61555 | 27265 | 30251 | 38487 | 107999 | NA | 75212 | 18222 | 14871 | 69709 | 141270 | 766475 |
| EPI_ISL_819335 | **B.1.5** | **V69S05** | 59287 | 119314 | 82406 | 84721 | 95784 | 122654 | 76572 | 100291 | 65861 | 67620 | 17841 | 76264 | 49487 | 18282 | 1036384 |
| EPI_ISL_819297 | **B.1.5** | **V69S13** | 115690 | 111134 | 117480 | 73151 | 128069 | 102058 | 133318 | 81248 | 91786 | 68668 | 35982 | 63523 | 52409 | 24938 | 1199454 |
| EPI_ISL_819344 | **B.1.5** | **V82S04** | 76258 | 70663 | 102169 | 15028 | 112223 | 33570 | 69235 | 56306 | 155310 | 38236 | 109038 | 60232 | 90667 | 107453 | 1096388 |
| EPI_ISL_819350 | **B.1.5** | **V82S06** | 55478 | 260781 | 124467 | 140955 | 268908 | 148584 | 112595 | 125893 | 169374 | 58084 | 95902 | 15425 | 79391 | 93688 | 1749525 |
| EPI_ISL_819298 | **B.1.5** | **V70S02** | 56164 | 58094 | 62756 | 38133 | 78899 | 55223 | 67951 | 45575 | 62095 | 40119 | 35383 | 10472 | 43375 | 34100 | 688339 |
| EPI_ISL_819355 | **B.1.5** | **V70S03** | 55428 | 65919 | 75835 | 44382 | 86904 | 66628 | 79013 | 52659 | 71394 | 41580 | 52007 | 13674 | 46286 | 38363 | 790072 |
| EPI_ISL_819349 | **B.1.1** | **V69S03** | 74190 | 90686 | 78070 | 64128 | 87225 | 80516 | 84226 | 66033 | 67171 | 64381 | 43646 | 56554 | 47715 | 19602 | 924143 |
| EPI_ISL_819351 | **B.1.1** | **V69S06** | 86507 | 96193 | 93692 | 75954 | 93329 | 88489 | 118136 | 79371 | 82397 | 77730 | 64881 | 104698 | 51431 | 54297 | 1167105 |
| EPI_ISL_819352 | **B.1.1** | **V69S07** | 53789 | 117433 | 91438 | 54310 | 113517 | 65910 | 89036 | 73434 | 51308 | 22418 | 19972 | 41298 | 42494 | 10149 | 846506 |
| EPI_ISL_819353 | **B.1.1** | **V69S08** | 64029 | 110201 | 76401 | 48962 | 85054 | 84962 | 121463 | 75768 | 50449 | 54072 | 55832 | 32290 | 27884 | 21100 | 908467 |
| EPI_ISL_819354 | **B.1.1** | **V69S09** | 68562 | 65086 | 66018 | 52557 | 66256 | 63164 | 76641 | 53316 | 60251 | 57254 | 45062 | 73022 | 38736 | 38502 | 824427 |
| EPI_ISL_819356 | **B.1.1** | **V69S10** | 56735 | 107504 | 62183 | 69428 | 65034 | 112265 | 56067 | 88889 | 48194 | 75519 | 26248 | 79421 | 38607 | 20179 | 906273 |
| EPI_ISL_819359 | **B.1.1** | **V69S11** | 56790 | 63012 | 65529 | 50924 | 67069 | 61533 | 73663 | 51476 | 62182 | 47690 | 43038 | 68962 | 34043 | 41939 | 787850 |
| EPI_ISL_819360 | **B.1.1** | **V69S12** | 63594 | 85570 | 70239 | 50582 | 87224 | 78022 | 82513 | 59684 | 49033 | 49791 | 19592 | 44140 | 35694 | 15708 | 791386 |
| EPI_ISL_1116590 | **B.1.177** | **V70S14** | 92249 | 79953 | 91515 | 56698 | 115622 | 83199 | 106053 | 71745 | 101242 | 60224 | 66658 | 19563 | 68132 | 49415 | 1062268 |
| EPI_ISL_1116569 | **B.1.177** | **V71S10** | 94032 | 75331 | 105336 | 82394 | 94474 | 94356 | 126573 | 79166 | 100955 | 70866 | 110737 | 113219 | 73477 | 98567 | 1319483 |
| EPI_ISL_1116591 | **B.1.177** | **V71S12** | 107971 | 99320 | 98066 | 81118 | 156318 | 119521 | 113663 | 100481 | 111096 | 92299 | 100102 | 75014 | 87324 | 24094 | 1366387 |
| EPI_ISL_1116585 | **B.1.177** | **V71S13** | 87571 | 81658 | 88880 | 85122 | 104026 | 92577 | 114823 | 78882 | 103392 | 70851 | 104989 | 89877 | 74011 | 65260 | 1241919 |
| EPI_ISL_1669361 | **B.1.177** | **V75S08** | 102187 | 69688 | 86358 | 74324 | 73905 | 72351 | 99389 | 64045 | 77889 | 73573 | 90015 | 113659 | 61058 | 101318 | 1159759 |
| EPI_ISL_1391101 | **B.1.177** | **V75S09** | 98727 | 71355 | 86490 | 67638 | 76774 | 68628 | 86742 | 59735 | 74888 | 71140 | 83632 | 89344 | 61441 | 90599 | 1087133 |
| EPI_ISL_1391138 | **B.1.177** | **V75S10** | 56959 | 126898 | 86203 | 68131 | 73630 | 93104 | 40564 | 80489 | 57164 | 57857 | 63051 | 32936 | 63406 | 107920 | 1008312 |
| EPI_ISL_1797511 | **B.1.177** | **V77S12** | 129584 | 94883 | 135667 | 102186 | 124546 | 98019 | 59507 | 94355 | 72530 | 98131 | 56515 | 81944 | 66420 | 110128 | 1324415 |
| EPI_ISL_1391158 | **B.1.177** | **V77S13** | 129078 | 129579 | 134781 | 119916 | 128995 | 121594 | 67562 | 109427 | 92664 | 110939 | 67729 | 83887 | 78597 | 122199 | 1496947 |
| EPI_ISL_1391140 | **B.1.177** | **V75S13** | 97697 | 82536 | 92963 | 65416 | 87265 | 70434 | 85185 | 58959 | 92027 | 69325 | 76386 | 53336 | 63049 | 81677 | 1076255 |
| EPI_ISL_1391141 | **B.1.177** | **V75S14** | 99420 | 58046 | 76605 | 80759 | 67026 | 66660 | 110095 | 65845 | 72605 | 71974 | 94027 | 119548 | 63456 | 109660 | 1155726 |
| EPI_ISL_1116562 | **B.1.1.7** | **V70S05** | 48748 | 105558 | 56303 | 73317 | 53698 | 95824 | 51023 | 85346 | 53964 | 84169 | 58711 | 30009 | 42629 | 62970 | 902269 |
| EPI_ISL_1116578 | **B.1.1.7** | **V70S06** | 61975 | 74786 | 83109 | 42006 | 79190 | 63200 | 86575 | 54562 | 82451 | 38610 | 60258 | 17486 | 43126 | 38333 | 825667 |
| EPI_ISL_1116564 | **B.1.1.7** | **V82S05** | 162545 | 161503 | 134202 | 104445 | 161271 | 118698 | 47759 | 155011 | 103832 | 116879 | 72540 | 31591 | 117758 | 126743 | 1614777 |
| EPI_ISL_1116558 | **B.1.1.7** | **V70S08** | 46021 | 79936 | 63161 | 54491 | 56045 | 74163 | 59725 | 58344 | 56724 | 37598 | 43732 | 59208 | 30129 | 72577 | 791854 |
| EPI_ISL_1116565 | **B.1.1.7** | **V70S09** | 117283 | 136708 | 148901 | 83795 | 127297 | 119410 | 155899 | 92441 | 130987 | 58091 | 104791 | 85632 | 64777 | 118114 | 1544126 |
| EPI_ISL_1116566 | **B.1.1.7** | **V70S10** | 86543 | 88873 | 119454 | 54675 | 107306 | 76502 | 120645 | 62927 | 110515 | 44677 | 86224 | 38493 | 56143 | 66178 | 1119155 |
| EPI_ISL_1116557 | **B.1.1.7** | **V70S11** | 77852 | 67512 | 87700 | 47367 | 81365 | 60843 | 91880 | 53362 | 81099 | 52884 | 75396 | 34601 | 53599 | 48707 | 914167 |
| EPI_ISL_1116561 | **B.1.1.7** | **V70S12** | 81255 | 77445 | 104561 | 52978 | 97169 | 69268 | 104971 | 58313 | 95787 | 51299 | 82467 | 34206 | 57294 | 54985 | 1021998 |
| EPI_ISL_1116580 | **B.1.1.7** | **V71S17** | 98738 | 98622 | 128069 | 86541 | 112337 | 96512 | 128661 | 87090 | 112919 | 78074 | 115796 | 98978 | 81528 | 71957 | 1395822 |
| EPI_ISL_1020573 | **B.1.351** | **V70S04** | 72348 | 64873 | 91867 | 24367 | 81902 | 66232 | 100713 | 54404 | 86327 | 38495 | 69864 | 35892 | 45601 | 54367 | 887252 |
| EPI_ISL_918301 | **B.1.351** | **V71S01** | 94060 | 69278 | 91836 | 46838 | 84602 | 79784 | 92247 | 71177 | 79240 | 75819 | 95594 | 109349 | 70046 | 58154 | 1118024 |
| EPI_ISL_1080829 | **B.1.351** | **V71S02** | 102017 | 88263 | 114009 | 63693 | 102344 | 105377 | 129810 | 95239 | 104585 | 89220 | 121661 | 138234 | 82233 | 96901 | 1433586 |
| EPI_ISL_1080831 | **B.1.351** | **V71S03** | 126774 | 112227 | 121361 | 52741 | 146134 | 113124 | 101878 | 104562 | 129937 | 113456 | 112764 | 94713 | 114031 | 18429 | 1462131 |
| EPI_ISL_2047723 | **B.1.351** | **V75S01** | 95969 | 88964 | 132128 | 32174 | 99186 | 76837 | 90809 | 64237 | 115487 | 60457 | 70689 | 52278 | 56596 | 100673 | 1136484 |
| EPI_ISL_2162254 | **B.1.351** | **V75S02** | 111422 | 73152 | 102696 | 43301 | 56846 | 75597 | 102546 | 66600 | 82771 | 73287 | 95858 | 115447 | 68431 | 113099 | 1181053 |
| EPI_ISL_1797493 | **B.1.351** | **V75S03** | 105295 | 66325 | 94406 | 36792 | 77637 | 68688 | 89947 | 59943 | 75203 | 72598 | 84142 | 94956 | 64221 | 100302 | 1090455 |
| EPI_ISL_1908823 | **B.1.351** | **V75S04** | 114593 | 65376 | 91465 | 44472 | 75788 | 74624 | 111387 | 69915 | 81091 | 83931 | 100441 | 141581 | 69299 | 121239 | 1245202 |
| EPI_ISL_2047794 | **B.1.351** | **V75S05** | 96018 | 79903 | 118486 | 32389 | 88113 | 71598 | 93870 | 58298 | 102233 | 59073 | 78216 | 72758 | 52117 | 93828 | 1096900 |
| EPI_ISL_2047795 | **B.1.351** | **V75S06** | 100703 | 71054 | 96787 | 41035 | 76579 | 70793 | 110059 | 65186 | 94460 | 72924 | 84971 | 122365 | 64775 | 112734 | 1184425 |
| EPI_ISL_1669413 | **B.1.351** | **V75S07** | 113441 | 80796 | 109746 | 50327 | 85647 | 81377 | 111431 | 71772 | 96841 | 83410 | 97483 | 129674 | 64785 | 123656 | 1300386 |
| EPI_ISL_2284919 | **B.1.617.2** | **V77S01** | 119007 | 107420 | 94612 | 87128 | 89161 | 79438 | 114885 | 76752 | 88201 | 91004 | 110772 | 138024 | 75481 | 122533 | 1394418 |
| EPI_ISL_2391926 | **B.1.617.2** | **V77S02** | 95069 | 113692 | 71151 | 109576 | 79502 | 118481 | 85582 | 112234 | 75176 | 117538 | 94922 | 81173 | 72021 | 149883 | 1376000 |
| EPI_ISL_2391913 | **B.1.617.2** | **V77S03** | 126946 | 104124 | 101821 | 85792 | 101288 | 80455 | 119764 | 73396 | 95613 | 86381 | 112642 | 137440 | 81413 | 120760 | 1427835 |
| EPI_ISL_2391912 | **B.1.617.2** | **V77S04** | 129324 | 94257 | 101482 | 105396 | 99484 | 81903 | 136317 | 85563 | 103748 | 94000 | 130509 | 180334 | 75787 | 127445 | 1545549 |
| EPI_ISL_2458145 | **B.1.617.2** | **V77S05** | 127310 | 123350 | 91094 | 88614 | 129171 | 105192 | 76662 | 90965 | 110769 | 85466 | 94254 | 37121 | 93630 | 124786 | 1378384 |
| EPI_ISL_2458160 | **B.1.617.2** | **V77S06** | 152233 | 122386 | 116582 | 98579 | 141701 | 94628 | 110785 | 114588 | 146335 | 115386 | 111752 | 46207 | 123005 | 150218 | 1644385 |
| EPI_ISL_2458155 | **B.1.617.2** | **V77S07** | 127562 | 105437 | 99323 | 90868 | 101342 | 86865 | 120929 | 80362 | 102846 | 95054 | 115646 | 136012 | 80203 | 128046 | 1470495 |
| EPI_ISL_2458152 | **B.1.617.2** | **V77S08** | 133210 | 87442 | 109150 | 99543 | 109529 | 97334 | 128660 | 89017 | 103503 | 102361 | 123923 | 148069 | 84285 | 138411 | 1554437 |
| EPI_ISL_2458146 | **B.1.617.2** | **V77S09** | 119879 | 3814 | 89013 | 86008 | 84519 | 76668 | 114782 | 73387 | 87308 | 86149 | 108721 | 141069 | 71577 | 122095 | 1264989 |
| EPI_ISL_2458140 | **B.1.617.2** | **V77S10** | 119023 | 104172 | 98905 | 103091 | 96040 | 96973 | 116462 | 93152 | 92359 | 95579 | 109465 | 153571 | 69958 | 142217 | 1490967 |
| EPI_ISL_2493136 | **B.1.617.2** | **V77S11** | 114216 | 112178 | 96877 | 75286 | 109385 | 77984 | 112202 | 67735 | 97345 | 70189 | 113053 | 108709 | 76760 | 90515 | 1322434 |
| EPI_ISL_8553063 | **B.1.1.529** | **V98S01** | 215083 | 50363 | 50935 | 52204 | 30943 | 26288 | 207752 | 52344 | 179780 | 60535 | 130987 | 107647 | 90268 | 70668 | 1325797 |
| EPI_ISL_8553046 | **B.1.1.529** | **V98S04** | 127521 | 54361 | 41715 | 48076 | 28965 | 22582 | 153097 | 47485 | 141799 | 49050 | 71462 | 83016 | 56468 | 52496 | 978093 |
| EPI_ISL_8552995 | **B.1.1.529** | **V98S06** | NA | NA | NA | NA | NA | NA | NA | 64471 | NA | NA | NA | 601959 | NA | 146433 | 812863 |
| EPI_ISL_8553066 | **B.1.1.529** | **V98S08** | 205574 | 62892 | 49625 | 56583 | 38583 | 21515 | 203306 | 60251 | 176638 | 75066 | 115182 | 126545 | 74983 | 92544 | 1359287 |
| EPI_ISL_8553061 | **B.1.1.529** | **V98S09** | 164260 | 81669 | 41237 | 66272 | 24640 | 35800 | 172116 | 65245 | 184487 | 87639 | 96229 | 118677 | 74305 | 56828 | 1269404 |
| EPI_ISL_8553227 | **B.1.1.529** | **V98S10** | 201647 | 72797 | 53350 | 69416 | 63899 | 42016 | 225428 | 66568 | 171154 | 69651 | 104167 | 122270 | 66596 | 88126 | 1417085 |
| EPI_ISL_8552957 | **B.1.1.529** | **V98S11** | 220546 | 77324 | 75747 | 39347 | 7524 | 7401 | 232379 | 52233 | 182470 | 49401 | 46008 | 93515 | 71264 | 45580 | 1200739 |
| EPI_ISL_3047229 | **B.1.1.529** | **V98S12** | 204075 | NA | 95594 | NA | 14510 | NA | 321757 | 10346 | 300759 | NA | 128224 | 69172 | 57570 | 34835 | 1236842 |
| EPI_ISL_8552968 | **B.1.1.529** | **V100S01** | 213904 | NA | 43499 | 8800 | 15406 | NA | 217555 | 28239 | 163603 | 40608 | 111880 | 83758 | 65725 | 55952 | 1048929 |
| EPI_ISL_7597244 | **B.1.1.529** | **V100S02** | 105358 | 87976 | 27609 | 75502 | 33266 | 42913 | 107815 | 67971 | 122359 | 74595 | 71000 | 123907 | 42749 | 95640 | 1078660 |
| EPI_ISL_855297 | **B.1.1.529** | **V100S03** | 165474 | 56187 | 47818 | 52130 | 20935 | 4787 | 169502 | 50740 | 156067 | 63385 | 96736 | 112955 | 59560 | 81352 | 1137628 |
| EPI_ISL_8553020 | **B.1.1.529** | **V100S04** | 140744 | 47205 | 31398 | 51218 | 10104 | 19793 | 121441 | 48812 | 109733 | 58671 | 76516 | 102058 | 56397 | 64355 | 938445 |
| EPI_ISL_8553025 | **B.1.1.529** | **V100S05** | 185094 | 57879 | 35339 | 60386 | 8002 | 11931 | 167187 | 57135 | 134314 | 70246 | 100325 | 125462 | 65118 | 84071 | 1162489 |
| EPI_ISL_8553259 | **B.1.1.529** | **V100S06** | 120095 | 45604 | 18474 | 48426 | NA | 4087 | 123649 | 67420 | 54731 | 84741 | 58859 | 130661 | 42048 | 56392 | 855187 |
| EPI_ISL_8553647 | **B.1.1.529** | **V100S07** | 160068 | 56203 | 36799 | 61014 | 33066 | 28661 | 161851 | 56007 | 125889 | 58282 | 93374 | 108485 | 57637 | 81282 | 1118618 |
| EPI_ISL_8553024 | **B.1.1.529** | **V100S08** | 120511 | 83652 | 27864 | 69560 | 8578 | 32540 | 119784 | 77481 | 99604 | 88942 | 84466 | 122901 | 69088 | 67877 | 1072848 |
| NA | **B.1.1.529** | **V100S09** | 114402 | 83614 | 48195 | 93550 | 35463 | 60451 | 214311 | 35713 | 70971 | 57261 | 59885 | 48270 | 32607 | 31737 | 986430 |
| NA | **B.1.1.529** | **V100S10** | 171299 | 43183 | 70243 | 37519 | 64158 | 26278 | 198722 | 33328 | 194431 | 40471 | 94220 | 65837 | 68023 | 48325 | 1156037 |
| NA | **B.1.1.529** | **V100S11** | 141047 | 84503 | 47786 | 72236 | 14957 | 71720 | 139915 | 54122 | 99967 | 68796 | 32298 | 55203 | 49317 | 24258 | 956125 |
|  |  |  | **A71** | **A72** | **A73** | **A74** | **A75** | **A76** | **A77** | **A78** | **A79** | **A80** | **A81** | **A82** | **A83** | **A84** | total reads |
|  |  | Total | 8757859 | 6720663 | 6417303 | 5012854 | 6819496 | 5352864 | 9121761 | 5730959 | 8101994 | 5453382 | 6105562 | 7076443 | 4811260 | 6044155 | **91526555** |

**Supplementary Table S3.** Comparison of coverage between amplicons. IQR=interquartile range. CV=coefficient of variation (also known as RSD=relative standard deviation).

| **Amplicon** | **Log(Reads) Median** | **CV** | **IQR** |
| --- | --- | --- | --- |
| **A71** | 5,021 | 0,035 | 0,202 |
| **A72** | 4,899 | 0,052 | 0,196 |
| **A73** | 4,891 | 0,039 | 0,210 |
| **A74** | 4,784 | 0,040 | 0,219 |
| **A75** | 4,840 | 0,071 | 0,228 |
| **A76** | 4,788 | 0,064 | 0,188 |
| **A77** | 5,037 | 0,035 | 0,172 |
| **A78** | 4,837 | 0,035 | 0,173 |
| **A79** | 4,993 | 0,034 | 0,193 |
| **A80** | 4,798 | 0,056 | 0,170 |
| **A81** | 4,870 | 0,043 | 0,242 |
| **A82** | 4,866 | 0,061 | 0,356 |
| **A83** | 4,788 | 0,027 | 0,156 |
| **A84** | 4,815 | 0,058 | 0,376 |

**Supplementary Table S4.** Minimum and maximum coverages, annotated number of patients with defective particles in amplicon A78 nt 1905 to 2260, aa636-aa753) (WithDef column) and percentage of patients with defective haplotypes per lineage.

| **Lineage** | **Count** | **MinCov** | **MaxCov** | **MedianCov** | **WithDef** | **Pctg** |
| --- | --- | --- | --- | --- | --- | --- |
| B.1.1 | 8 | 51476 | 88889 | 69733.5 | 2 | 25.0 |
| B.1.5 | 9 | 45575 | 215520 | 81248.0 | 3 | 33.3 |
| B.1.177 | 11 | 58959 | 109427 | 78882.0 | 6 | 54.5 |
| Alpha | 9 | 53362 | 155011 | 62927.0 | 0 | 0.0 |
| Beta | 11 | 54404 | 104562 | 66600.0 | 0 | 0.0 |
| Delta | 11 | 67735 | 114588 | 85563.0 | 0 | 0.0 |
| Omicron | 19 | 10346 | 77481 | 54122.0 | 4 | 21.1 |

**Supplementary Table S5.** Primers used to amplify the N07 region (see Andres et al[2] for further details).

| **N07 primers** | **Spike region** | **Primer MN908947.3Wuhan-Hu-1** | **Size (nts)** | **Tm (ºC)** | **G/C(%)** | **Product size** |
| --- | --- | --- | --- | --- | --- | --- |
| Forward | 1798-1827 | 5' - CAGTGTTATAACACCAGGAACAAAT - 3' | 25 | 61.8 | 36 | 367 nts |
| Reverse | 2148-2175 | 5' - ATTTGTGGGTATGGCAATAGAGTTA - 3' | 25 | 63.2 | 36 |  |

**Supplementary Table S6.** Coverage and frequency of deletions and defective genomes obtained in samples amplified in parallel using the ARTIC (amplicon A78) and N07 (region N07[2]) protocols. delNTS=number of deleted nucleotides. Hpl=haplotype (read with the same sequence).

|  | **N07** | | | | | **A78** | | | | |
| --- | --- | --- | --- | --- | --- | --- | --- | --- | --- | --- |
|  | **delNTS** | **Reads (Hpl)** | **Total reads per sample** | **Freq per amplicon** | **Defective genomes** | **delNTS** | **Reads, Hpl** | **Total reads A78** | **Freq per amplicon** | **Defective genomes** |
| **V69S03** | 0 | 0 | 237449 | 0 | NO | 0 | 0 | 66033 | 0 | NO |
| **V69S06** | 0 | 0 | 251265 | 0 | NO | 0 | 0 | 79371 | 0 | NO |
| **V69S09** | 28 | 398 | 159700 | 0.25% | YES | 24 | 574 | 53316 | 1.08% | NO |
|  |  |  |  |  |  | 28 | 125 |  | 0.23% | YES |
|  |  |  |  |  |  |  | **699** |  |  |  |
| **V69S10** | 0 | 0 | 202720 | 0 | NO | 0 | 0 | 88889 | 0 | NO |
| **V69S11** | 0 | 0 | 158182 | 0 | NO | 0 | 0 | 51476 | 0 | NO |
| **V69S12** | 0 | 0 | 218836 | 0 | NO | 0 | 0 | 59684 | 0 | NO |
| **V69S13** | 2 | 399 | 242697 | 0.16% | YES | 9 | 399 | 81248 | 0.49% | YES |
|  |  |  |  |  |  | 13 | 229 |  | 0.28% | YES |
|  |  |  |  |  |  | 28 | 257 |  | 0.32% | YES |
|  |  |  |  |  |  | 29 | 226 |  | 0.28% | YES |
|  |  |  |  |  |  | 38 | 229 |  | 0.28% | YES |
|  |  |  |  |  |  | 41 | 198 |  | 0.24% | YES |
|  |  |  |  |  |  |  | **1538** |  |  |  |
| **V70S02** | 2 | 4007 | 240491 | 1.67% | YES | 18 | 366 | 45575 | 0.8% | YES |
|  |  |  |  |  |  | 28 | 408 |  | 0.9% | YES |
|  |  |  |  |  |  | 35 | 396 |  | 0.87% | YES |
|  |  |  |  |  |  |  | **1170** |  |  |  |
| **V70S03** | 0 | 0 | 219193 | 0 | NO | 0 | 0 | 52659 | 0 | NO |
| **V70S04** | 0 | 0 | 222674 | 0 | NO | 0 | 0 | 54404 | 0 | NO |
| **V70S05** | 0 | 0 | 254207 | 0 | NO | 0 | 0 | 85346 | 0 | NO |
| **V70S06** | 0 | 0 | 217385 | 0 | NO | 0 | 0 | 54562 | 0 | NO |
| **V82S05** | 0 | 0 | 232196 | 0 | NO | 0 | 0 | 155011 | 0 | NO |
| **V70S08** | 0 | 0 | 234294 | 0 | NO | 0 | 0 | 58344 | 0 | NO |
| **V70S09** | 0 | 0 | 209088 | 0 | NO | 0 | 0 | 92441 | 0 | NO |
| **V70S10** | 0 | 0 | 214608 | 0 | NO | 0 | 0 | 62927 | 0 | NO |
| **V70S11** | 0 | 0 | 216140 | 0 | NO | 0 | 0 | 53362 | 0 | NO |
| **V70S12** | 2 | 286 | 215553 | 0.13% | YES | 0 | 0 | 58313 | 0 | NO |
|  | 7 | 318 |  | 0.15% | YES |  |  |  |  |  |
|  |  | **604** |  |  |  |  |  |  |  |  |
| **V70S14** | 0 |  | 243104 | 0 | NO | 0 | 0 | 71745 | 0 | NO |
| **V71S10** | 22 | 385 | 165063 | 0.23% | YES | 22 | 204 | 79166 | 0.26% | YES |
|  | 28 | 685 |  | 0.41% | YES | 28 | 400 |  | 0.51% | YES |
|  | 29 | 320 |  | 0.19% | YES | 29 | 206 |  | 0.26% | YES |
|  |  | **1390** |  |  |  |  | **810** |  |  |  |
| **V71S12** | 0 | 0 | 250266 | 0 | NO | 0 | 0 | 100481 | 0 | NO |
| **V71S13** | 23 | 472 | 221521 | 0.21% | YES | 23 | 225 | 78882 | 0.29% | YES |
|  | 28 | 1033 |  | 0.47% | YES | 28 | 302 |  | 0.38% | YES |
|  | 29 | 274 |  | 0.12% | YES |  | **527** |  |  |  |
|  |  | **1779** |  |  |  |  |  |  |  |  |
| **V71S17** | 0 | 0 | 232695 | 0 | NO | 0 | 0 | 87090 | 0 | NO |
| **V71S01** | 2 | 472 | 231343 | 0.21% | YES | 0 | 0 | 71177 | 0 | NO |
| **V71S02** | 0 | 0 | 224642 | 0 | NO | 0 | 0 | 95239 | 0 | NO |
